# Inference of selective forces on house mouse genomes during secondary contact in East Asia

**DOI:** 10.1101/gr.278828.123

**Published:** 2024-03

**Authors:** Kazumichi Fujiwara, Shunpei Kubo, Toshinori Endo, Toyoyuki Takada, Toshihiko Shiroishi, Hitoshi Suzuki, Naoki Osada

**Affiliations:** 1 Mouse Genomics Resource Laboratory, National Institute of Genetics, Mishima 411-8540, Japan;; 2 Graduate School of Information Science and Technology, Hokkaido University, Sapporo 060-0814, Japan;; 3 Integrated BioResource Information Division, RIKEN BioResource Research Center, Tsukuba 305-0074, Japan;; 4 RIKEN BioResource Research Center, Tsukuba 305-0074, Japan;; 5 Graduate School of Environmental Science, Hokkaido University, Sapporo 060-0810, Japan

## Abstract

The house mouse (*Mus musculus*), which is commensal to humans, has spread globally via human activities, leading to secondary contact between genetically divergent subspecies. This pattern of genetic admixture can provide insights into the selective forces at play in this well-studied model organism. Our analysis of 163 house mouse genomes, with a particular focus on East Asia, revealed substantial admixture between the subspecies *castaneus* and *musculus*, particularly in Japan and southern China. We revealed, despite the different level of autosomal admixture among regions, that all Y Chromosomes in the East Asian samples belonged to the *musculus*-type haplogroup, potentially explained by genomic conflict under sex-ratio distortion owing to varying copy numbers of ampliconic genes on sex chromosomes, *Slx* and *Sly*. Our computer simulations, designed to replicate the observed scenario, show that the preferential fixation of *musculus*-type Y Chromosomes can be achieved with a slight increase in the male-to-female birth ratio. We also investigated the influence of selection on the posthybridization of the subspecies *castaneus* and *musculus* in Japan. Even though the genetic background of most Japanese samples closely resembles the subspecies *musculus*, certain genomic regions overrepresented the *castaneus*-like genetic components, particularly in immune-related genes. Furthermore, a large genomic block (∼2 Mbp) containing a vomeronasal/olfactory receptor gene cluster predominantly harbored *castaneus*-type haplotypes in the Japanese samples, highlighting the crucial role of olfaction-based recognition in shaping hybrid genomes.

The house mouse (*Mus musculus*), an important laboratory animal, is commensal to humans and has spread worldwide owing to human activities such as colonization, agricultural expansion, and trade (Gündüz et al. 2001; Geraldes et al. 2008; Searle et al. 2009a,b; Gabriel et al. 2011; Macholán et al. 2012; Jones et al. 2013; Li et al. 2021). The house mouse shows high morphological and genetic diversity, with three main subspecies recognized: *M. musculus castaneus*, *M. musculus musculus*, and *M. musculus domesticus* (Boursot et al. 1996; Salcedo et al. 2007; Phifer-Rixey et al. 2020; Fujiwara et al. 2022a). These subspecies diverged ∼180–500 thousand years ago (kya) and underwent secondary contact after dispersing from their native habitat around South Asia (Boursot et al. 1993; Din et al. 1996) with asymmetric hybrid incompatibility; the subspecies *domesticus* shows relatively strong hybrid incompatibility with the other two subspecies (White et al. 2011, 2012). Despite partial reproductive isolation between subspecies, genetic studies have revealed a nonnegligible amount of gene flow, as well as occasional identification of hybrid genotypes (Orth et al. 1998; Ďureje et al. 2012; Fujiwara et al. 2022a). Studying the genetic differentiation and hybridization patterns in *M. musculus* thus provides a unique opportunity to gain insights into human history and the genetic architecture of hybridization among genetically distinct subspecies of a well-studied model organism.

In East Asia, two major subspecies have been widely observed: The subspecies *castaneus* is distributed in southern China and Taiwan, and the subspecies *musculus* is distributed in northern China, the Korean Peninsula, and the Russian Far East (Din et al. 1996; Jing et al. 2014; Fujiwara et al. 2022a). Another subspecies, *M. musculus molossinus*, has been recognized in the Japanese archipelago, where researchers have shown through genetic analysis that it is derived from a hybrid between the subspecies *castaneus* and *musculus* (Yonekawa et al. 1980, 1982, 1988; Sakai et al. 2005; Takada et al. 2013), whereas most wild Japanese house mice predominantly have the genetic background of the subspecies *musculus* (Fujiwara et al. 2022a). Mitochondrial haplogroups representing the subspecies *castaneus* are mainly distributed in northern Japan, whereas mitochondrial haplogroups representing the subspecies *musculus* are observed in southern Japan (Terashima et al. 2006; Kuwayama et al. 2017; Li et al. 2021), leading to the hypothesis that the subspecies *castaneus* was introduced from southern China to the Japanese archipelago and *musculus* was introduced from the Korean Peninsula along with the wet-rice field cultivation system (Terashima et al. 2006; Kuwayama et al. 2017). This hypothesis would be similar to the well-known model of the origin of the modern Japanese population, called the dual structure model by Hanihara (1991). The dual structure model assumes that the genetic makeup of the modern Japanese population has been composed of both indigenous Jomon hunter-gatherers and newly migrated Yayoi rice farmers (Hanihara 1991). Recent genetic analyses of modern and ancient human genome studies have revealed that the Jomon originated from one of the basal East Asian populations (McColl et al. 2018; Kanzawa-Kiriyama et al. 2019; Gakuhari et al. 2020; Osada and Kawai 2021). Despite the progress in genomic research in humans, studies into the demographic history of *M. musculus* in East Asia using a genome-wide data set have not been performed.

Although the genetic diversity of maternally inherited mitochondrial haplotypes in wild house mice has been well studied, natural variation in the paternally inherited Y Chromosome has remained largely unknown, except for a few examples (Morgan and Pardo-Manuel de Villena 2017). Recent experimental studies have discovered X- and Y-linked loci that are crucial for hybrid incompatibility, in which copy number variation plays a significant role in determining hybrid incompatibility and sex-ratio distortion (SD). Previous studies have shown that Y-linked *Sly* suppresses postmeiotic expression of X- and Y-linked genes in sperm (Ellis et al. 2011; Cocquet et al. 2012). A copy number increase in *Sly* results in male-biased progenies, whereas a copy number increase in X-linked *Slx* counteracts this (Scavetta and Tautz 2010; Cocquet et al. 2012). Morgan and Pardo-Manuel (2017) showed that the subspecies *musculus* tended to have higher *Sly*/*Slx* copy numbers than other subspecies, but their analyzed samples were biased toward wild-derived inbred lines (Morgan and Pardo-Manuel de Villena 2017). Therefore, the analysis of copy number variations of *Sly*/*Slx* and their natural distribution range in wild samples would provide insight into how the SD alleles affect the genetic differentiation of mouse subspecies.

The selective forces on the formation of hybrid genomes are also an important issue in evolutionary biology research. With the advancement of genome sequencing technology, many genome-scale studies have been conducted to examine the effects of natural selection under interspecific or intersubspecific gene flow. In particular, adaptive gene introgression, in which beneficial alleles cross the boundary of populations and spread rapidly to another population, has attracted the interest of many researchers (Song et al. 2011; Pardo-Diaz et al. 2012; Huerta-Sánchez et al. 2014; Enard and Petrov 2018). The distinctive genetic characteristics of wild Japanese house mice would provide a great opportunity to investigate the effects of selection during hybridization. Having the major genetic background of the subspecies *musculus*, the preference for the genomic region of *castaneus* ancestry in the Japanese population leaves a similar signature to that of adaptive introgression and is a robust signal of natural selection during hybrid genome formation.

In this study, we analyze 163 high-coverage whole-genome sequences of *M. musculus*, including newly sequenced samples primarily from Japan, to quantify the pattern of genetic admixture among the samples. By contrasting the population differentiation patterns of autosomal and sex-linked genomic regions, we infer the evolutionary history of the two East Asian subspecies populations. We also analyze Japanese samples to identify specific regions of the genome where genetic components from the subspecies *castaneus* are more prevalent, even though the majority of the Japanese samples have a genetic background from the subspecies *musculus*. Focusing on the evolutionary history of *M. musculus* in East Asia, this study provides new insights into how this commensal animal has shaped its genetic traits through secondary contact mediated by human activities.

## Results

### Genome-wide pattern of differentiation in East Asia

In this study, we sequenced the genome of 37 wild house mice mainly collected from Japan, with an average coverage of 26.3 (Supplemental Table S1). Those data were merged with a previously published data set that included *Mus spretus*, yielding the genotypes of 170 samples. After filtering, 133,886,237 autosomal biallelic single-nucleotide variants (SNVs) were used for the initial population study (Supplemental Method 1).

Principal component analysis (PCA) was performed on all *M. musculus* samples (Supplemental Fig. S1). The results were in high agreement with those previously reported by Fujiwara et al. (2022a). Three major genetic components corresponding to subspecies *castaneus*, *domesticus*, and *musculus* were observed. Most of the East Asian samples aligned on the *castaneus–musculus* cline, which means that the genetic characteristics of the East Asian samples can be modeled by different degrees of admixture between the subspecies *castaneus* and *musculus*. Genetic clustering was performed using ADMIXTURE software (Alexander et al. 2009) assuming three ancestral populations (*K* = 3). The results yielded three genetic components corresponding to the three main subspecies, consistent with the PCA results (Supplemental Fig. S2).

**Figure 1.**
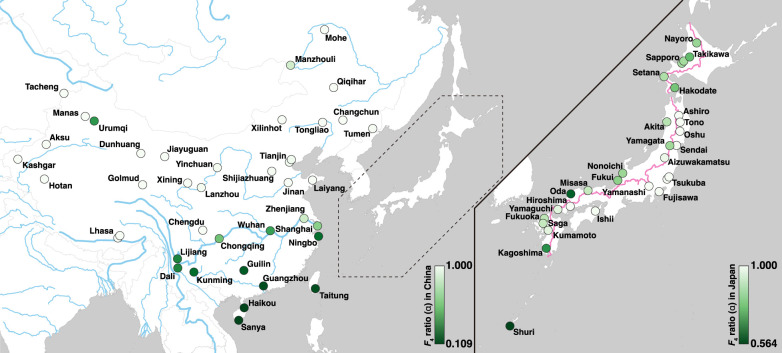
Estimated ratio of *musculus/castaneus* ancestry in East Asia. The colors of circles indicate the *musculus*/*castaneus* ancestry of samples at each collection site: (*left*) East Asian samples except for the Japanese archipelago, (*right*) Japanese archipelago samples. The *musculus* ancestry and *castaneus* ancestry of each sample are represented by a range of colors from white to green, with the stronger green color indicating higher *castaneus* ancestry. The range of the ancestry ratio is indicated by the color bar in each panel. The pink line in *right* panel represents the central watershed in the Japanese archipelago.

To quantify the degree of genetic admixture in each sample, the *f*_4_-ratio value developed by Patterson et al. (2012) was calculated for each sample (see Methods), assuming that the East Asian samples were derived from an admixture of two unknown lineages that shared ancestry with the Korean *musculus* and Indian *castaneus* populations. The estimated percentage of *musculus* ancestry, expressed as α, varied from 0.109 to one among the East Asian samples, as shown in Figure 1. In the Japanese archipelago, all samples had α-values greater than 0.564, and the mean value of α was 0.878 (Fig. 1). The samples from the Sea of Japan side have lower α-values than those from the Pacific Ocean side. When the sample from Okinawa (a subtropical island) was excluded and the other samples were divided into two classes according to the central watershed of Japan, the Pacific-side samples had significantly higher α-values than the Sea of Japan-side samples (*P* = 0.0002, Mann–Whitney *U* test). In China, α-values were close to one in the northern and western regions but were much lower in southern China (Fig. 1). In the southern Chinese samples, the average α-value was 0.295, indicating that the genetic background of southern Chinese samples is mostly derived from the subspecies *castaneus*.

### Genetic differentiation at mitochondrial genome and sex chromosomes

We compared genetic differentiation patterns at autosomal, X-chromosomal, Y-chromosomal, and mitochondrial loci. We observed generally less mixing of subspecies genomes on the X Chromosome; namely, there was less *castaneus* ancestry in the Japanese population and less *musculus* ancestry in the southern Chinese population on the X Chromosomes. The estimated α-values on the X Chromosome ranged from 0.317 to one (average α: 0.860) for Japanese samples and from 0.034 to 0.952 (average α: 0.207) for southern Chinese samples, showing that the X Chromosome has a low level of introgression into the predominant genome. The differences in α-value between autosomes and X Chromosomes were significant in both the Japanese (*P* = 0.0008) and southern Chinese (*P* = 0.0134) populations (Wilcoxon signed-rank test).

Mitochondrial genomes of the samples were reconstructed through de novo assembly (for details, see Supplemental Method 3), and their phylogeographic patterns were then compared with those observed in the Y Chromosomes. The genealogies of the mitochondrial and Y-chromosomal loci inferred by the maximum-likelihood method (Supplemental Method 2) are shown in Supplemental Figures S3 and S4, respectively. The Y-chromosomal locus formed three distinct haplogroups corresponding to the three major subspecies, whereas the mitochondrial locus showed five major haplogroups. In addition to the three mitochondrial haplogroups representing the subspecies *castaneus*, *domesticus*, and *musculus*, samples from Madagascar and Nepal formed distinct mitochondrial haplogroups, as previously shown (Fujiwara et al. 2022a,b).

**Figure 2.**
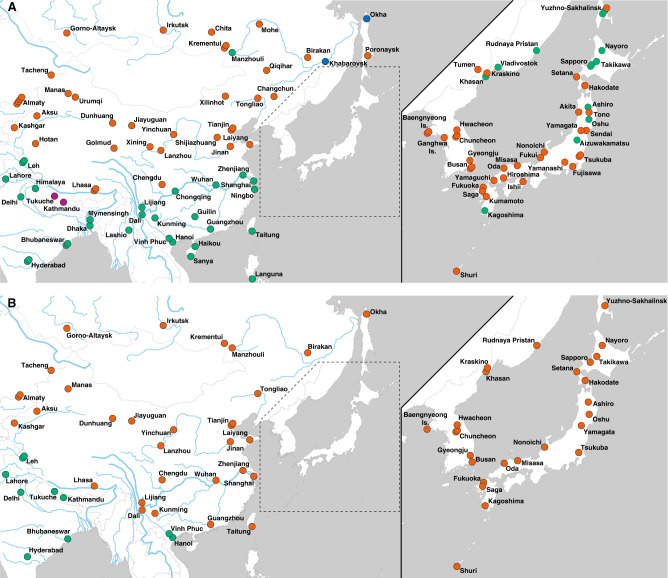
Geographic distribution of the subspecies *castaneus* and *musculus* haplotypes of mitochondrial and Y-chromosomal loci. *A* and *B* show the mitochondrial and Y-chromosomal haplotypes, respectively. The colors of circles indicate subspecies haplotypes based on genotyping: *castaneus*-type (green), *domesticus*-type (blue), *musculus*-type (red), and the geographically confined group of Nepalese-type (purple).

The geographic distribution of the subspecies *castaneus-* and *musculus-*type haplotypes was markedly different between mitochondrial and Y-chromosomal loci (Fig. 2). The distribution of mitochondrial haplotypes was consistent with previous findings (Fig. 2A); in the Japanese archipelago, *castaneus-* and *musculus-*type mitochondrial haplotypes are distributed in the northern and southern regions of mainland Japan, respectively. In China, the distribution of mitochondrial haplotypes was clearly divided around the Yangtze River basin (or divided at the Qinling Mountains), with *musculus*-type haplotypes in northern China and *castaneus*-type haplotypes in southern China. However, all Y-chromosomal haplotypes in East Asia were assigned exclusively to *musculus*-type haplotypes (Fig. 2B). The Y Chromosome of the reference genome (C57BL/6J) also had a *musculus*-type haplotype, and it was closely related to the haplotype of Japanese samples from the Tohoku area (Supplemental Fig. S4), supporting the idea that the Y Chromosome of classical inbred strains originated from Japanese house mice (Japanese fancy mouse) (Bishop et al. 1985; Nagamine et al. 1992; Tucker et al. 1992; Takada et al. 2013).

**Figure 3.**
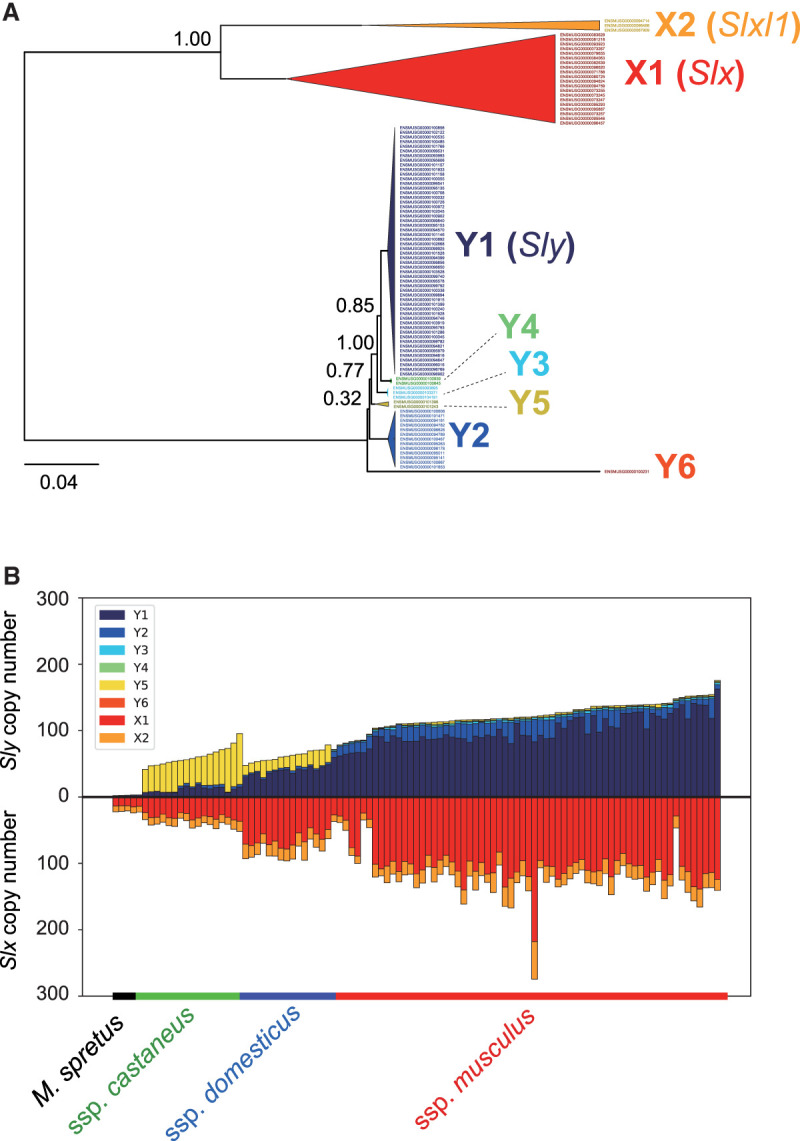
Copy number evolution of *Sly* and *Slx*. (*A*) The phylogenetic tree of *Sly*/*Slx* genes on the mouse reference genome, GRCm38. Bootstrap values are described at each node. (*B*) Estimated copy numbers of *Sly* and *Slx* for each sample. Different colors represent the copy numbers of different clusters. The samples were aligned according to the subspecies group of Y Chromosome haplotypes first and then sorted ascendingly by the total copy numbers of *Sly* within the groups.

We hypothesized that the dominance of the *musculus*-type Y Chromosome in East Asia is caused by a distorted sex ratio caused by a dosage imbalance in the *Sly*/*Slx* genes; *Sly* and *Slx* are gametologs under X–Y sexual conflict and are highly duplicated genes (more than 100 copies). Previous studies have shown that *musculus*-type Y Chromosomes encompass more *Sly* copies and that the subspecies *musculus* has more copies of *Slx* on the X Chromosome (Morgan and Pardo-Manuel de Villena 2017). Estimating the copy numbers of *Sly* and *Slx*, however, is challenging owing to the presence of highly similar but distinct classes of repetitive sequences in the genome. To tackle this, we first aligned the paralogous sequences of the *Sly* and *Slx* loci present in the reference genome and used these alignments to reconstruct a phylogenetic tree. The reconstructed tree showed two clusters for *Slx*, which correspond to *Slx* (X1) and *Slxl1* (X2), and six clusters for *Sly* (Y1–Y6), with one cluster (Y1) being the most abundant in the reference genome and including the canonical *Sly* (Fig. 3A). We then estimated copy numbers for each cluster based on the short-read depth of cluster-specific SNV alleles (see Methods). We observed that different subspecies-level Y haplotypes harbored different patterns of *Sly* clusters (Fig. 3B). Cluster Y5 was the major cluster in the *castaneus*-type haplotypes, whereas cluster Y1 was the major cluster in the *domesticus*-type and *musculus*-type Y haplotypes. Particularly, *castaneus*- and *domesticus*-type Y haplotypes had low *Sly* copy numbers, whereas *musculus*-type Y haplotypes had high copy numbers among the three haplogroups. In contrast, the copy number ratios of *Slx* and *Slxl1* were mostly consistent among samples. As shown in Figure 3B, Supplemental Figure S5, and a previous study, *Sly* and *Slx* copy numbers in the same male individuals were highly correlated (*P* < 10^−22^, Spearman's rank correlation test) (Morgan and Pardo-Manuel de Villena 2017).

It is remarkable that the southern Chinese population predominantly shares a genetic background with the subspecies *castaneus* yet exclusively carries Y Chromosomes from the subspecies *musculus*. To explore the selective pressures driving this distinct pattern of Y Chromosome haplotypes in southern China, we conducted Wright–Fisher model simulations. The model assumes that a small fraction of the subspecies *musculus* individuals with longer haplotypes of the SD locus on the sex chromosomes (corresponding to *Sly* and *Slx*) migrate to the subspecies *castaneus* population at a constant rate, *Nm*, where *N* and *m* represent effective population size and the migratory fraction of individuals per generation, respectively. An F_1_ hybrid between a subspecies *musculus* male and a subspecies *castaneus* female tend to have more male offspring than female offspring owing to the segregation distortion of X/Y Chromosomes in a male germline. Detailed methods and results are documented in Supplemental Note 1, Supplemental Figures S6 through S10, and Supplemental Table S2.

Briefly, without assuming mutations on copy numbers at the SD locus, the simulations showed that the introgressed Y Chromosomes with longer SD haplotypes (corresponding to the *musculus-*type *Sly*/*Slx* haplotypes) spread in the recipient population within 0.1*N* generations even with a 10%–20% increase in the male-to-female birth ratio (Supplemental Fig. S6A). Without the SD effect, there is little chance for the introgressed alleles to be fixed in the recipient population within 0.1*N* generations with the assumed migration rate (*Nm* = 1). The X Chromosomes with longer SD haplotypes also eventually fix in the recipient population; however, the fixation of longer SD haplotypes was in general much faster on the Y Chromosomes than on the X Chromosomes because we assume that SD occurs on the male germline.

We also investigate the effect of hybrid incompatibility between different genomes, including the incompatibility between autosomes, between autosomes and X Chromosomes, and between autosomes and mitochondrial genomes (Presgraves 2008; Bonnet et al. 2017; Healy and Burton 2020; Fraïsse and Sachdeva 2021). In particular, the incompatibility between autosomes and X Chromosomes would be highly likely given that we observed less introgression of X Chromosomes than autosomes in the two hybrid populations, Japanese and southern Chinese populations, with different directions of introgression. The difference in fixation time between the introgressed Y and X Chromosomes becomes longer with the effect of the X-chromosomal hybrid incompatibility (XHI). When the effect of XHI is sufficiently strong, the introgression of X Chromosomes with longer SD haplotypes is completely suppressed (Supplemental Fig. S7C).

### Selection during admixture in the Japanese population

To identify genomic regions affected by natural/sexual selection during intersubspecific admixture, we focused on samples from the Japanese archipelago and searched for genomic regions highly enriched in *castaneus*-ancestry haplotypes. Following the results presented in Figure 1, we estimated the values of α, representing the proportion of *musculus*-like genomic components, for each nonoverlapping 20-kb-long window of the autosomal genome.

To discern the neutral distribution of *castaneus*-enriched genomic blocks within the Japanese house mouse population, we used fastsimcoal2 software (Excoffier et al. 2013) and inferred demographic parameters from our sample set (for details, see Supplemental Note 2; Supplemental Figs. S9, S10; Supplemental Table S3). Using the estimated demographic parameters, we executed 200,000 coalescent simulations to draw a distribution of α under neutrality. From our results, we identified 2655 outlier windows. These windows, which fell below the 5% threshold of the neutral distribution, encompassed the protein-coding regions of 842 distinct genes. A comprehensive list of these genes can be found in Supplemental Table S4.

We first tested the correlation between the *castaneus*-enriched windows with the gene density and recombination rate. The *castaneus*-enriched windows in the Japanese samples showed significant depletion of genes (*P* < 10^−5^, chi-square test), as well as a significantly lower recombination rate (*P* < 10^−12^, Mann–Whitney *U* test), compared with the genomic background (Supplemental Fig. S11). We further performed functional enrichment analysis on the 842 candidate genes using Metascape software (Zhou et al. 2019). A total of 20 gene categories were found to be enriched in the list (*P*-values < 0.01), with the complete list of significantly overrepresented functional categories presented in Supplemental Figure S12. The enriched term list contained a variety of gene categories, but notably, immune-related gene categories such as immunoglobulin production (GO:002377), antibody-dependent cellular cytotoxicity (GO:0001788), and herpes simplex virus 1 infection (mmu05168) were overrepresented. Indeed, our screening accurately identified the retroelement-like Friend virus susceptibility protein 1 (*Fv1*) gene, an antiviral factor (Best et al. 1996), as a *castaneus*-ancestry-enriched gene in Japan. Previous studies have shown that the *Fv1*^*b*^ allele, which lacks the 1.3-kb deletion commonly found in the subspecies *domesticus* and *musculus*, is observed with high frequency in the subspecies *castaneus* and Japanese samples (Boso et al. 2021). We confirmed that the deletion started from Chr 4: 147,870,288 (GRCm38) and was 1.2 kb in length, resulting in truncation of the C-terminal region of *Fv1* (Supplemental Method 4). The allele frequencies for the deletion were 0.92, 0.64, and 0.16 in the subspecies *domesticus*, *musculus*, and *castaneus*, respectively. The allele frequency in the Japanese samples was 0.02, showing that almost no Japanese house mice harbor the deletion.

**Figure 4.**
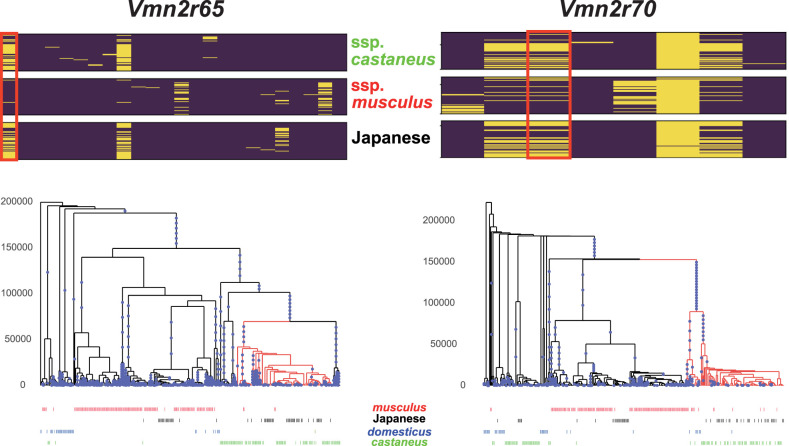
Haplotype structure of nonsynonymous SNVs and the genealogies of wild house mouse *Vmn2r65* and *Vmn2r70.* (*Top*) The segregation pattern of nonsynonymous SNVs. The sites in the red rectangles represent the focal sites. (*Bottom*) The inferred genealogies around the focal sites. The blue dots indicate mutations on branches. The branches with the derived mutations at the focal sites are colored in red. The labels of the samples are shown at the *bottom* of the tree.

**Figure 5.**
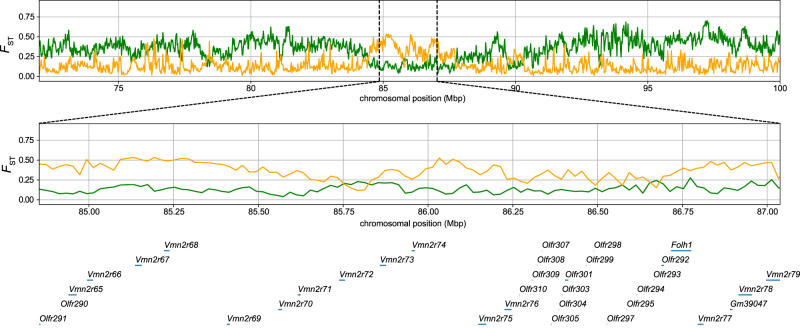
Distribution of *F*_ST_ in 20-kb windows on Chromosome 7. The yellow and green lines indicate *F*_ST_ between the Japanese and subspecies *musculus* samples and *F*_ST_ between the Japanese and subspecies *castaneus* samples, respectively. The *bottom* panel shows the pattern in the olfactory/vomeronasal genes in this cluster (Chr 7: 84,853,553–87,037,968, GRCm38).

We also observed that 23 vomeronasal receptor genes (*Vmn1r* and *Vmn2r*), including one pseudogene, were present in the gene list. In the enrichment analysis, one particular category, detection of chemical stimulus involved in sensory perception (GO0050907), showed the strongest bias (Supplemental Fig. S12). These *castaneus*-enriched vomeronasal receptor genes were scattered across different olfactory/vomeronasal genomic clusters on the mouse genome, with most genes located in a large cluster on Chromosome 7, region Chr 7: 84,853,553–87,037,968, containing 17 olfactory and 15 vomeronasal receptors. Of the 23 vomeronasal receptor genes identified in our genomic scan, 12 were located in the cluster. For illustration purposes, we present the segregation pattern of SNVs in the *Vmn2r65* and *Vmn2r70* genes residing in this cluster (Fig. 4). These examples distinctly show that the Japanese samples show a closer genetic affinity to the subspecies *castaneus* than to the subspecies *musculus* in these regions. To confirm that the estimated low α in the region was not caused by errors in statistical inference, we computed the *F*_ST_ between Japanese and subspecies *musculus* samples (*F*_ST-MUS/JPN_) and the *F*_ST_ between Japanese and subspecies *castaneus* samples (*F*_ST-CAS/JPN_). The subspecies *castaneus* and *musculus* samples were assigned from the result of the ADMIXTURE analysis. We plotted window-averaged *F*_ST-CAS/JPN_ and *F*_ST-MUS/JPN_ values across the region, including Chr 7: 84,853,553–87,037,968 (Fig. 5). As expected, outside of this region, the *F*_ST-MUS/JPN_ values were consistently smaller than the *F*_ST-CAS/JPN_ values, supporting the notion that Japanese samples were generally *musculus*-like. However, the pattern was completely reversed within the olfactory/vomeronasal clusters, indicating an unusual pattern of genetic admixture in this region. We also estimated genealogies around the target nonsynonymous SNVs of the *Vmn2r* genes using RELATE software (Supplemental Method 5; Speidel et al. 2019). The genealogies encompassing *Vmn2r65* and *Vmn2r70* showed that the *castaneus*-type Japanese alleles coalesced with the other *castaneus*-type alleles after the split of the subspecies *castaneus* and *musculus*, which was estimated to occur ∼200 kya from our data (Fig. 4).

## Discussion

### Genetic differentiation and inferred history of *M. musculus* in East Asia

The widespread coexistence of the subspecies *castaneus* and *musculus* in East Asia suggests that they formed a broad hybrid zone following their initial migration from their homeland. Previous studies have inferred migration scenarios primarily based on phylogeographical patterns of mitochondrial genomes (Jing et al. 2014; Li et al. 2021). We present a combined view from autosomal, mitochondrial, X-chromosomal, and Y-chromosomal loci result in Supplemental Note 3.

### Spread of *musculus*-type Y Chromosomes in East Asia

In this study, we uncovered natural variation in the Y Chromosomes of house mice across Eurasia, identifying distinct clusters corresponding to major subspecies. All Y Chromosomes in East Asian samples were of the *musculus*-type, which could be explained by various mechanisms such as natural/sexual selection (Veltsos et al. 2008; Petr et al. 2020), male-biased migration (Tosi et al. 2003), and genetic drift. Genomic conflict between X and Y Chromosomes offers a plausible explanation for such biased transmission, as suggested in species such as *Drosophila* (Branco et al. 2013) and the great apes (Nam et al. 2015). In our specific case, the *Sly*/*Slx* system is likely a major contributor, for which SD is well validated by experimental evidence.

In the *Sly*/*Slx* system, the subspecies *musculus* possesses longer, and thus potentially more advantageous, haplotypes for both the X and Y Chromosomes. This genetic advantage should theoretically expedite the introgression of both chromosome types but at different magnitudes. Our computer simulations indicated faster fixation of *musculus*-type Y Chromosomes compared with *musculus*-type X Chromosomes, even in the absence of other factors. However, when considering hybrid incompatibility between genomes, particularly between autosomes and X Chromosomes as well as autosomes and mitochondrial genomes, the rate of introgression for *musculus*-type X Chromosomes was significantly reduced (Supplemental Fig. S7; Supplemental Table S2). Previous research has highlighted the significance of both autosomal–X chromosomal (Presgraves 2008; Fraïsse and Sachdeva 2021) and autosomal–mitochondrial hybrid incompatibilities (Bonnet et al. 2017; Healy and Burton 2020), supported by both experimental and theoretical evidence. We thus propose that these factors also play a crucial role in the pronounced difference in introgression patterns between X and Y Chromosomes observed in southern China.

### Post-admixture natural selection in the Japanese archipelago

Detecting the signatures of natural selection during genetic admixture is an important yet challenging topic in evolutionary biology. This challenge arises because, following admixture or introgression, it is typically the pre-existing alleles (i.e., standing variation) rather than new mutations that are subject to selection. Traditional methods for detecting selective sweeps generally focus on hard sweeps driven by new mutations. In contrast, post-admixture positive selection often manifests as soft sweeps, which are statistically more difficult to detect (e.g., Hermisson et al. 2017). The unique genetic composition of Japanese house mice presents an excellent opportunity to robustly investigate selection signatures, showing the complexity of evolutionary processes at play.

To establish thresholds for identifying *castaneus*-enriched genomic regions within Japanese populations, we estimated demographic parameters using fastsimcoal2 (see Supplemental Note 2; Supplemental Fig. S9). Despite the agreement between expected and observed site frequency spectra after parameter optimization in the Japanese population (Supplemental Fig. S10), caution is advised when interpreting parameters derived from fastsimcoal2 because of the model's inherent limitations. Consequently, the representation of *castaneus*-enriched windows may not be entirely accurate. Nonetheless, a robust and discernible pattern among these windows suggests the influence of natural selection. Notably, we observed a significant reduction in gene density and recombination rates within *castaneus*-enriched windows, indicating the role of negative selection in purging introgressed alleles, aligning with observations from studies on human–Neanderthal admixtures (Sankararaman et al. 2014). Although these windows are outliers within a neutral framework, many *castaneus*-type alleles in these regions likely increase in frequency owing to genetic drift. In particular, a decrease in local effective population size owing to reduced recombination rates enhances the likelihood of introgressed alleles achieving high frequency within a recipient population. Nevertheless, the prevalence of immune-related and olfactory-related genes within these windows points toward their positive selection, hinting at their adaptive significance despite the overarching negative selection pressure.

Genes involved in host defense mechanisms were significantly enriched in the outlier loci. In the case of humans, several studies have shown evidence of adaptive introgression from archaic to modern humans at immune-related loci (Racimo et al. 2015; Enard and Petrov 2018). For example, we identified *Irgm1* and *Irgm2*, which are GTPases involved in the interferon signaling pathway, in our candidate list. In particular, *Irgm2* has been shown to be responsible for defense against *Toxoplasma*, and several *castaneus*-type tightly linked nonsynonymous SNVs were common among the Japanese samples (Supplemental Figs. S13, S14). In another example, the *castaneus*-ancestry-enriched regions contained six cathepsin genes on Chromosome 13. Two of them, *Ctsj* and *Ctsr*, are exclusive to the adult placenta and may be responsible for the process of viral infection. Although the roles of *Ctsj* and *Ctsr* in viral infection have yet to be shown, maternal–fetal viral transmission might be a critical factor during the process of hybrid formation. Alternatively, these placental genes may be responsible for the compatibility between the genotypes of mother and offspring.

In addition to these genes, vomeronasal receptors showed a strongly biased pattern of segregation. These receptors are expressed in the vomeronasal organ and form a large receptor family involved in vomeronasal chemosensation (Wynn et al. 2012). They recognize a wide variety of chemical cues, such as pheromones from different sexes and predator odors (Dulac and Torello 2003). In some vomeronasal receptor genes, most Japanese samples harbor *castaneus*-type nonsynonymous alleles. Although certain vomeronasal receptor genes are highly differentiated between house mouse subspecies and could serve as markers for discrimination (Wynn et al. 2012), our genealogical analysis revealed that the target genes might have frequently crossed subspecies boundaries (Fig. 4). Furthermore, we noted a concentration of mutations along the branch that differentiates the *castaneus*- and *musculus*-type haplotypes of *Vnm2r70* (Fig. 4). This clustering suggests the influence of positive selection in driving the diversification of these haplotypes. It is thus possible that some vomeronasal alleles become adaptive in other subspecies and quickly spread following introgression. Frequent introgression of olfactory and other chemosensory receptor genes has been observed in the analysis between the subspecies *domesticus* and *musculus* (Staubach et al. 2012; Janoušek et al. 2015).

The identified vomeronasal receptors were primarily clustered in a region on mouse Chromosome 7, Chr 7: 84,853,553–87,037,968. This entire ∼2-Mbp region displayed a strongly biased pattern of *castaneus* ancestry (Fig. 5). Previous extensive studies revealed that *Vmn2r* genes in this cluster respond to cues from conspecific mating partners (Isogai et al. 2011). Detailed studies, such as gene replacement experiments, will elucidate how different vomeronasal receptor alleles contribute to the formation of natural house mouse hybrids in the future.

In this study, we have elucidated the genomic landscape of wild house mice in East Asia. In East Asian house mouse populations, which experienced secondary contact after the subspecies diverged, we have inferred the effects of natural/sexual selection and genomic conflict from the observed pattern of genetic structure at various genomic loci. These findings pave the way for future studies on genetic admixtures between different house mouse subspecies and provide insight into the genetic architecture of hybrid genomes.

## Methods

### Genomic samples

We sequenced the whole genomes of 37 *M. musculus* samples and used mitochondrial DNA sequences published in previous studies (Moriwaki et al. 1986; Suzuki et al. 2004, 2013; Terashima et al. 2006; Nunome et al. 2010; Kodama et al. 2015; Kuwayama et al. 2017). We combined these data with the global sample data set used in our prior research (Fujiwara et al. 2022a,b). A detailed list of these samples can be found in Supplemental Table S1. In total, our analysis included 163 *M. musculus* and seven *M. spretus* samples. The method for SNV genotyping is presented in Supplemental Method 1.

### Population genetics analysis

We conducted PCA on 163 *M. musculus* samples to investigate the population structure. This analysis was performed using smartpca from the Eigensoft software with default parameters, except that outliers were not excluded (Patterson et al. 2006).

To further analyze population structure and the admixture, we used ADMIXTURE with the number of clusters *K* = 3 for the 163 *M. musculus* samples. Before admixture inference, we pruned SNVs under linkage disequilibrium (LD) using PLINK 1.9 (Purcell et al. 2007) with the option “‐‐indep-pairwise 50 5 0.5.” In downstream analyses, the majority of ancestral proportions determined by ADMIXTURE analysis define the subspecies of each sample.

To quantify the fraction of genomes contributed by the subspecies *musculus* (α) in the East Asian samples, we estimated *f*_4_-ratio statistics (Patterson et al. 2012). The estimation of *f*_4_ statistics was performed using the “patterson_d” function in the Scikit-allel (https://github.com/cggh/scikit-allel) Python package. Assuming the source of *musculus* genomic components was more closely related to the Korean *musculus* samples than to Kazakh *musculus* samples, we estimated the α-value for sample X according to Equation 1:

(1) $$\hat{\alpha }=\frac{\;f_{4}(\mathrm{KAZ},\;\mathrm{SPR};X,\;\mathrm{IND})}{\;f_{4}(\mathrm{KAZ},\;\mathrm{SPR};\mathrm{KOR},\;\mathrm{IND})}$$

where KAZ, SPR, KOR, and IND represent the samples from Kazakhstan (five samples), *M*. *spretus* (seven samples), Korea (nine samples), and India (two samples from Leh), respectively. In this scenario, sample X acquires a portion of its genome from the subspecies *musculus* (α) and the remaining proportion (1 − α) from the subspecies *castaneus.* Note that although the *f*_4_-ratio statistics allow us to infer the mixing proportions of an admixture event, it cannot determine the direction of introgression. The model is also presented in Supplemental Figure S9. In principle, α is equal to or smaller than one, but it may slightly exceed one because of statistical fluctuation. When α > 1, we set α to one. Additionally, we estimated the value of α for each 20-kb-length window for all Japanese samples. To remove unreliably estimated values, we excluded windows with fewer than 100 SNVs. Both the denominator and numerator of the right side of Equation 1 are expected to be positive, considering that the Japanese and Korean samples are genetically closer to the subspecies *musculus* (KAZ samples) than to the subspecies *castaneus* (IND samples). However, a small fraction of windows showed negative denominators and/or numerators as a result of statistical errors, introgression, or ancestral polymorphisms. Such windows were filtered out from the following analysis. *F*_ST_ was estimated using the “hudson_fst” function in Scikit-allel.

### Copy number estimation of *Sly/Slx*

We downloaded all of the paralogous sequences of *Sly* (ENSMUSG00000101155) and *Slx* (ENSMUSG00000095063) on the sex chromosomes, including introns, from Ensembl Release 102. The sequences were then aligned using MAFFT (Katoh et al. 2002; Katoh and Standley 2013). Following the alignment, we reconstructed a phylogenetic tree using MEGA X software (Kumar et al. 2018), using a maximum-likelihood method with the general time-reversible (GTR) model. From this alignment, we identified tag-SNV sites with cluster-specific alleles.

We started by mapping the short reads from samples to either *Sly* or *Slx* genomic sequences, incorporating 1000-bp-length flanking sequences for both ends. The copy numbers for each cluster were then estimated by counting the depth of cluster-specific alleles at the tag-SNV sites, using the pileup format files generated from the SAMtools mpileup command. To filter out results from repetitive sequences that might complicate our analysis, we focused solely on sites with a mappability score of one (Supplemental Method 1), calculated using autosomal sequences and either *Sly* or *Slx* sequences. To ensure the accuracy and relevance of our results, a filter was applied to exclude sites that showed a depth smaller than one-third or larger than three times the median. Finally, we estimated the copy number for each sample from the mean depth, which was then divided by half the average depth of the whole genome for the sample.

## Data access

All raw short-read sequencing data generated in this study have been submitted to the DNA Data Bank of Japan (DDBJ) DDBJ Sequence Read Archive (DRA; https://www.ddbj.nig.ac.jp/dra/index-e.html) database under accession number PRJDB16017. All complete mitochondrial sequences generated in this study have also been submitted to the DDBJ (https://getentry.ddbj.nig.ac.jp/top-j.html) under accession numbers LC772928–LC772964. The data sets, parameterization files, and scripts required to reproduce the analysis were submitted as Supplemental Data and deposited in the Dryad digital repository (https://datadryad.org/) under doi:https://doi.org/10.5061/dryad.9p8cz8wnb.

## Supplementary Material

Supplement 1

Supplement 2

Supplement 3

Supplement 4

Supplement 5

Supplement 6

Supplement 7
