# Supplementary material for: Inference of selective forces on house mouse genomes during secondary contact in East Asia
Source: Genome Res. 2024 Mar;34(3):366–75. doi: 10.1101/gr.278828.123 (PMC11067880; doi:10.1101/gr.278828.123)
Supplement: Supplement 1 [file Supplemental_Documents.pdf]

Supplemental Materials for

Inference of selective force on house mouse genome during secondary contact in East Asia

Kazumichi Fujiwara, Shunpei Kubo, Toshinori Endo, Toyoyuki Takada, Toshihiko Shiroishi, Hitoshi Suzuki, Naoki Osada

Table of Contents:

1 **Supplemental Note 1**: Computer simulations for the introgression of X-chromosomal, Y-chromosomal, and mitochondrial genomes under sex-ratio distortion

5 **Supplemental Note 2**: Demographic inference and computer simulations for admixture

7 **Supplemental Note 3**: Dispersal model of *M. musculus* in East Asia

9 **Supplemental Method 1**: Mapping and genotype calling

11 **Supplemental Method 2**: Y Chromosome re-sequencing

12 **Supplemental Method 3**: Mitochondrial genome assembly

13 **Supplemental Method 4**: Deletion detection from the sequence data

14 **Supplemental Method 5**: Construction of genome-wide genealogies using RELATE

15 **Reference**

Supplemental Note 1

Computer simulations for the introgression of X-chromosomal, Y-chromosomal, and mitochondrial genomes under sex-ratio distortion

Introduction

Our simulations replicated the scenario observed in southern China, where house mouse populations primarily possess the genetic background of the subspecies *castaneus*, yet their Y Chromosomes are exclusively of the subspecies *musculus*-type. We postulated that the *castaneus*-type Y Chromosomes in the southern Chinese population were displaced by *musculus*-type Y Chromosomes, driven by sex-ratio distortion (SD) linked to the *Sly*/*Slx* loci. In the model, individuals possessing genotypes that cause SD continually migrate from another population. To determine the feasibility of this exclusive Y Chromosome replacement within a plausible range of parameters and timeframes, we executed simulations under varying conditions.

Model

We modeled a Wright–Fisher diploid population A of size N, which consists of males and females with the XY sex-determination system. Population A receives migrants from population B at a rate of m (fraction of migrants in the recipient population) per generation. Assuming that populations A and B are already well differentiated genetically, for each individual in population A, we consider five key genetic parameters: sex, a genomic fraction derived from population B (G_{A}), genotypes at the SD loci on the X (G_{XSD}) and/or Y (G_{YSD}) Chromosomes, genotypes at the two X-chromosomal hybrid incompatibility (XHI) loci (G_{XHI1} and G_{XHI2}) adjacent to the SD locus, and mitochondrial genotype (G_{M}). We assumed that the SD and XHI loci are recombined at a rate of r per generation. In the following definitions, we assigned the value 0 and 1 to the original alleles in populations A and B, respectively.

We assumed that the autosomal genome would experience a sufficient amount of recombination. The feature of the offspring's genome, G_{A}, is therefore scored by an average of the parental values. G_{A} of the F1 hybrid between an individual from non-admixed populations A and B would thus become 0.5, and G_{A} of the backcrossed hybrid in population A would be 0.25. The genetic distance between parental genomes at autosomal locus, d_{AA}, was defined as the absolute difference between the G_{A} values of a father and a mother. Similarly, the genetic distance between autosomes and X Chromosomes, d_{XA}, was defined as the average of the difference (G_{XHI}) between G_{A} and G_{XHI1} and between G_{A} and G_{XHI2}. Since a female offspring has two X Chromosomes, G_{XHI} in females was averaged over the two chromosomes. The genetic distance between autosomal and mitochondrial genomes, d_{MA}, and the genetic distance between X and Y Chromosomal genomes, d_{XY}, were defined

in a similar way.

The SD locus is located on both X and Y Chromosomes. We assumed that the SD locus has copy number polymorphisms and that allele type 1 in population B has a higher copy number than allele type 0 in population A. In a male germline, a sex chromosome that has an SD allele with a higher copy number than the other sex chromosome is assumed to have a higher probability of transmission to the offspring. The parameter α denotes the level of SD when X and Y Chromosomes mismatch in male gametes; when the allele on the Y Chromosome is 1 and the allele on the X Chromosome is 0, the male-to-female ratio of offspring would be α, and vice versa.

The effect of hybrid incompatibility was evaluated for each reproduced offspring. Assuming the multiplicative effect of hybrid incompatibility between autosomal genomes, between the X Chromosome and autosomal genomes, and between the mitochondrial and autosomal genomes, the fitness of offspring, w, is defined according to equation (1):

$$w = exp[-R_{\mathrm{AA}}d_{\mathrm{AA}} - R_{\mathrm{XA}}d_{\mathrm{XA}} - R_{\mathrm{MA}}d_{\mathrm{MA}} - R_{\mathrm{XY}}d_{\mathrm{XY}}] \quad (1)$$

where R_{AA}, R_{XA}, R_{MA}, and R_{XY} represent amplitude factors for each effect.

Simulations

We developed a Python script for an individual-based population genetics simulation, which consists of N_{m} males and N_{f} females in population A. N_{m} and N_{f} may not always be equal, but their sum, N, remains constant. Each generation in the simulation has two phases.

In the first phase, recombination between each XHI locus and SD locus on the X Chromosomes in females is randomly assigned. Random variables are drawn from the Poisson distribution with a mean of $2rN_{\mathrm{f}}$, and the specified number of recombination events is randomly allocated to the pool of X Chromosomes in females. If mutations are considered, they are assigned to each genome during this phase in a similar manner.

In the second phase, mating pairs are randomly selected from population A. However, there is a probability of m that one mate will be replaced by a migrant from population B. The offspring's sex ratio is determined by the father's genotype, as described in the previous section. After selecting mating pairs, the offspring's fitness is evaluated. A random variable is drawn from a uniform distribution U(0, 1). If the random variable is higher than w, the individual is discarded, and a new mating pair is chosen until N offspring are produced for the next generation. This process is repeated for an adequate number of generations.

Results

We performed simulations using various parameter settings and present results with $N = 2{,}000$ for up to 200 ($0.1N$) generations. The number of generations is sufficient, considering that house mice have a population size in the order of 100,000–1,000,000, and we take into account the admixture events occurring around these 10,000 ($0.1N$) generations. We also fixed the migration rate $Nm = 1$. At this migration rate, we expect that introgressed alleles would not easily reach fixation under neutrality. We initially ignored recombination between the SD and XHI loci ($r = 0$) and discuss its effect below. In all simulations, we considered 1,000 instances.

We initially assessed the required intensity of SD for the rapid fixation of introgressed X and Y Chromosomes, examining whether the introgressed Y Chromosomes fix more rapidly than the X Chromosomes. By varying the value of α while setting all incompatibility parameters (R_{AA}, R_{XA}, R_{MA}, and R_{XY}) to 0, we examined the fixation rates of introgressed X and Y Chromosomes over $0.1N$ generations. The results indicated that a 10%–20% increase in the male-to-female birth ratio suffices for the rapid fixation of introgressed Y Chromosomes (Supplemental Figure S6A). Conversely, the fixation of introgressed X Chromosomes consistently lagged behind that of Y Chromosomes because of the design of our model, where the SD always occurs in male gametes. An increase of α to 40%–60% led to the fixation of introgressed X Chromosomes within $0.1N$ generations (Supplemental Figure S6A), but this fixation typically occurred later than that of the Y Chromosomes. Trajectories of allele frequencies in population A with $\alpha = 1.5$ are shown in Supplemental Figure S7A. In simulations of the reverse scenario, where X and Y Chromosomes with short haplotypes introgressed into a population with long X and Y haplotypes, the fixation of introgressed X and Y Chromosomes did not occur, aligning with expectations.

We then examined the impact of fertility reduction caused by SD. This reduction can be considered as incompatibility between X and Y Chromosomes in males. We set the male-to-female SD parameter, α, to 1.5 and varied the value of R_{XY}. The results, depicted in Supplemental Figure S6B, show that R_{XY} has a minimal effect until it reaches above 0.5. An R_{XY} value of 0.5 corresponds to a fertility reduction of 39%, indicating that even with a 50% increase in SD, the rapid fixation of introgressed Y Chromosomes can still occur despite a relatively high level of fertility reduction.

Although the above results clearly showed the advantage of introgressed Y Chromosomes over introgressed X Chromosomes in the recipient population without the influence of additional factors, we further evaluated the effect of hybrid incompatibility between different genomes because of the accumulating evidence of hybrid incompatibility loci between genomes. In addition, we have observed less introgression of X Chromosomes in southern China and Japan. By changing various hybrid incompatibility parameters (R_{AA}, R_{XA}, and R_{MA}) with $\alpha = 1.5$, $R_{XY} = 0$, and $r = 0$ (no recombination

between SD and XHI loci), we first investigated the effect of hybrid incompatibility due to a mismatch in the autosomal genomes (R_{AA} = 1, R_{XA} = 0, and R_{MA} = 0). The parameter set corresponds to a fitness reduction of F1 hybrids to 39%. We found that the level of hybrid incompatibility would certainly reduce the rate of introgression, but the fixation probability of introgressed alleles was still high with the selective advantage of introgressed X and Y Chromosomes (Supplemental Table S2). The trajectories of allele frequencies in population A are shown in Supplemental Figure S7B. The effect of the XHI locus was incorporated into the next case (R_{AA} = 0, R_{XA} = 1, and R_{MA} = 0). The X Chromosomal effect on hybrid incompatibility greatly reduced the fixation rate of introgressed X Chromosomes but did not change the fixation rate of introgressed Y Chromosomes (Supplemental Table S2). The trajectories of allele frequencies in population A are shown in Supplemental Figure S7C. We also incorporated the effect of mitonuclear incompatibility, where introgressed mitochondrial genomes are incompatible with the genomic background of recipients. Fixation rates of introgressed alleles in the case of R_{AA} = 0, R_{XA} = 0, and R_{MA} = 0 are presented in Supplemental Table S2 and the trajectories are shown in Supplemental Figure S7D.

We also investigated the effects of recombination between the SD and XHI loci on genomic introgression. Our findings reveal that even with a high recombination rate, the rate of fixation for introgressed X Chromosomes remains significantly reduced in scenarios involving autosome-X Chromosome incompatibility (Supplemental Figure S8). This outcome is attributed to the fact that recombination among X Chromosomes only occurs within female germlines, leading to an efficient elimination of introgressed X Chromosomes before the SD and XHI loci can be separated through recombination. Furthermore, our model posits that the SD locus is situated between two distinct XHI loci, diminishing the likelihood of the SD locus becoming unlinked from both XHI loci within a single generation.

Supplemental Note 2

Demographic inference and computer simulations for the admixture

Demographic parameter estimation

To illustrate the distribution of *castaneus*-enriched genomic blocks in the Japanese population under neutrality, we inferred demographic parameters for the five population samples (*M. spretus*, SPR; Kazakhstan, KAZ; Korea, KOR; India (Leh), IND; Japan, JPN) using Fastsimcoal2 software (Excoffier et al. 2013). The total number of samples used for the analysis was 52. The model is presented in Supplemental Figure S6. For simplicity, we assumed that each population size remained constant unless a population split or admixture occurred, that migration rates were symmetric, and that a single pulse admixture event took place during the establishment of the Japanese population. We also assumed that the admixture forming the Japanese population occurred 3,000 generation ago, with the effects of this assumption discussed later in the text.

We generated joint site frequency spectrum data with minor allele frequencies for the five populations using the same samples employed in calculation of the f_4 ratio (α), which estimates the genomic proportion of *musculus*-derived alleles in the Japanese samples. In addition to the mappability filter, we filtered our SNVs using the threshold of "ExcessHet>30", marked by GATK software (Van der Auwera et al. 2002).

The limitations of our genotyping pipeline meant that the monomorphic non-variant sites in the genome were not specified. To address this, we estimated the number of these sites. This estimation was essential to scale the parameters accurately. Our mappability mask identified 1,764,828,698 autosomal sites that passed the filtering process. Out of the 52 samples, we opted for the sites where all samples had been genotyped, leading to a reduction in polymorphic sites by a factor of 0.878. In addition, on the basis of the H1KG project, we postulated that 93% of the autosomal genomes were accessible (http://ftp.1000genomes.ebi.ac.uk/). By combining these data, the total analyzed sites amounted to 1,440,533,483.

We assumed a mutation rate of 5.9×10^{-9} and a generation time of 1 year, and repeated the maximum-likelihood estimation procedure 300 times, selecting the parameter sets that demonstrated the highest likelihood. We also conducted parametric bootstrap resampling 100 times to capture the 95% confidence interval. Details on the estimated population parameters can be found in Supplemental Table S3.

Following the optimization of parameters, we evaluated the fit of these parameters to the observed data. The log maximum-likelihood value post-optimization was -263,231,275, compared with the highest possible log-likelihood of -258,320,847. Although this deviation is notable, a comparison of

the expected and observed folded site frequency spectra within the Japanese population revealed a close match across all frequencies, with an exception at the frequency of 0.5 (see Supplemental Figure S10). This discrepancy at the 0.5 frequency could be attributed to our exclusion of SNVs that appeared heterozygous in many samples, applied by the ExcessHet>30 filter.

Computer simulations

We generated 20-kbp-length genomic fragments corresponding to the samples using the maximum-likelihood estimators in Supplemental Table S3, with a recombination rate of 1×10^{-8} per site per generation, and estimated α for 100,000 repetitions. We assigned P-values to the observed data using this distribution. When we did not fix the admixture timing for the Japanese population and estimated the timing using Fastsimcoal2, we obtained a slightly earlier admixture event estimate of > 5,000 generations ago, which was a much earlier timepoint than the assumed value. We are uncertain whether this deviation is due to our simplified model and assumptions; however, the older admixture event causes the distribution of α to skew toward higher values, making our test more conservative.

Supplemental Note 3

Dispersal model of M. musculus in East Asia

The pattern of autosomal and mitochondrial differentiation supports that the subspecies *musculus* reached East Asia through the northern side of the Himalayas (Li et al. 2021; Fujiwara et al. 2022b), similar to the route of Paleolithic human migration from central/western Eurasia to East Asia (Osada and Kawai 2021). Its migration coincided with the transmission of wheat in the Late Neolithic period from west to east and foxtail and broomcorn millets in the Early to Middle Neolithic periods from east to west Eurasia (Betts et al. 2014; Leipe et al. 2019). The migration route of the subspecies *castaneus* from North India to southern China is less clear, but it is likely that this migration was associated with rice cultivation. Whether japonica rice (*Oryza sativa japonica*) and indica rice (*Oryza sativa indica*) were established independently remains controversial, but they experienced considerable gene flow after the initial domestication event, suggesting that there was human activity linking the two regions during the Early Neolithic period (Huang et al. 2012; Choi et al. 2017). The spread of the subspecies *castaneus* in Chinese and Southeast Asian regions was probably caused by the spread of rice cultivation.

Archaeological findings highlight three primary Neolithic cultural hubs in East Asia: the Yellow River basin and West Liao River basin, centered around foxtail and broomcorn millet farming, and the Yangtze River basin, known for its rice cultivation. Recent ancient human genome sequencing revealed that genetic differentiation between the people in these centers was stronger than that of modern Chinese human populations (Ning et al. 2020; Yang et al. 2020). The current distribution of the subspecies *musculus* and *castaneus* corresponds to the genetic makeup of ancient humans and is associated with the spread of certain types of crop cultivation in East Asia. However, the degree of admixture between the subspecies *musculus* and *castaneus* appears to be smaller than that between human populations in northern and southern China at non-Y Chromosome loci, reflecting the restricted gene flow of house mice.

Differentiation of M. musculus in the Japanese archipelago

Both our previous and current analyses show that the genomic components of the subspecies *musculus* are predominant in samples from the Japanese archipelago (Fujiwara et al. 2022a). The strong genetic relatedness of Japanese and Korean samples at all genomic loci strongly supports the idea that the subspecies *musculus* was introduced to the Japanese archipelago by rice farmers with irrigation facilities (Yayoi people) who migrated through the Korean Peninsula beginning in 1,000 BCE (Fujio 2017). However, a recent study analyzing seed impressions in pottery revealed that early migrants from the Korean Peninsula to the Japanese archipelago during the Final Jomon and Initial Yayoi periods used both rice and millet (Endo and Leipe 2022). This suggests that the subspecies

musculus may have reached the Japanese archipelago alongside millet farming, rather than rice farming.

Given that 80%–90% of the modern Japanese genome is derived from continental migrants, human migration from continental Asia to the Japanese archipelago was extensive and continuous (Hanihara 1991; Kanzawa-Kiriyama et al. 2019; Cooke et al. 2021; Osada and Kawai 2021). Considering that all *musculus*-type mitochondrial and Y-chromosomal haplotypes in the Japanese samples are derived from one or two haplotypes from the Korean *musculus* lineage, it is likely that there were a limited number of subspecies of *musculus* migrants. The subsequent population explosion of the subspecies *musculus* indicates its success in the rice-crop-oriented society in Japan (Yonekawa et al. 1988). However, how and when the subspecies *castaneus* reached the Japanese archipelago remain unclear. Future studies using ancient samples may help to answer these questions.

Our analysis reveals a clear pattern of differentiation between the coastline of the Japanese archipelago. Recent large-scale genome analyses of the modern Japanese population have not shown such a distinct trend (Watanabe et al. 2021). In addition, the majority of animal species in the Japanese archipelago show genetic clustering between north-east and south-west regions (e.g., (Tsuchiya 1974; Kakioka et al. 2012; Okamoto and Hikida 2012; Tominaga et al. 2015; Dufresnes et al. 2016; Kato et al. 2020; Ito et al. 2021). We propose that this unusual pattern of differentiation was shaped based on anthropogenic reasons, particularly shipping trades. In the 18th century, Japan developed highly organized shipping trade routes along the Sea of Japan and Pacific Ocean coastlines. Considering that the subspecies *castaneus* mainly inhabited northern Japan, as evidenced by mitochondrial genomes, travel by sea may have facilitated the spread of genetic components of *castaneus* from northern Japan to the Sea of Japan coast. Although we do not have direct evidence to support the hypothesis, it would be worthwhile to test this in future studies using additional samples, including ancient samples.

Supplemental Method 1

Mapping and genotype calling

We sequenced the genomes of the 37 samples using the DNBSEQ platform (100- or 150-bp-length paired-end). These newly sequenced whole genome samples were the samples used primarily for mitochondrial analysis in previous studies. To assess the quality of the reads, FastQC (Andrews 2010) and MultiQC (Ewels et al. 2016) were employed for verification and visualization. The bwa-mem algorithm, specifically using the "-M" option (Li and Durbin 2009), facilitated the alignment of all raw reads to the GRCm38 (mm10) reference genome of the house mouse. PCR duplicate reads were flagged using the samblaster program with the "-M" option (Faust and Hall 2014). We used GATK4 HaplotypeCaller with the "-ERC GVCF" option (McKenna et al. 2010) for the initial calling of raw single nucleotide variants (SNVs) and insertions/deletions (indels). Subsequently, the genomic variant call format (gVCF) files from all newly sequenced samples and previously sequenced samples, including public datasets, were consolidated using the CombineGVCFs function, followed by a joint variant calling across all samples via the GenotypeGVCFs function. The GATK4 Variant Quality Score Recalibration (VQSR), which integrates machine learning to differentiate between true and false variants based on known variants, was applied to the raw SNVs and indels. For the VQSR process, we used the "mgp.v3.snps.rsIDdbSNPv137.vcf.gz" and "mgp.v3.indels.rsIDdbSNPv137.vcf.gz" files from the Sanger Institute's web server (ftp://ftp-mouse.sanger.ac.uk/REL-1303-SNPs_Indels-GRCm38/) as training sets for SNVs and indels, respectively. Additionally, hard-filtered SNV data were included in the training dataset. The criteria for hard-filtering SNVs were QD < 2.0; FS > 60.0; MQ < 40.0; MQRankSum < -12.5; and ReadPosRankSum < -8.0. For indels, the HARD-filtering parameters were QD < 2.0; FS > 200.0; InbreedingCoeff < -0.8; ReadPosRankSum < -20.0; and SOR > 10.0. We considered SNVs and indels within the top 90% tranche of the reliable training datasets as true-positive variants for further analysis. To ensure accurate mapping to the GRCm38 reference genome of house mouse, SNVs passing VQSR were additionally filtered based on mappability scores computed using GenMap (Pockrandt et al. 2020), with the parameters "-K 30" and "-E 2," focusing on sites with a mappability value of 1. Variant annotation and effect estimation on genes were conducted using SnpEff and SnpSift (Cingolani et al. 2012a; Cingolani et al. 2012b), with the "GRCm38.101" annotation dataset (ftp://ftp.ensembl.org/pub/release-101/gtf/mus_musculus/). Furthermore, we used ShapeIt4 software (Delaneau et al. 2019) to estimate phased haplotypes, incorporating the genetic map dataset (Liu et al. 2014). We used the RefSeq sequences NC_005089 and NC_205952 as the mitochondrial references for *M. musculus* and *M. spretus*, respectively. To determine the sex of the 37 samples, we analyzed the coverage of reads on the sex chromosomes using samtools depth, quantifying the coverage for each sample at non-pseudoautosomal regions of the X and Y Chromosomes following mappability filtering. The coverage ratios between the X and Y

Chromosomes exhibited a clear bimodal distribution, which allowed us to classify the samples as male or female based on their respective ranges.

Supplemental Method 2

Y Chromosome re-sequencing

We re-sequenced the Y Chromosomes using raw read data obtained by whole genome sequencing. The list of male samples that were used for Y genotyping is shown in Supplemental Table S1. The SNVs and Indels were called by GATK4 (McKenna et al. 2010) HaplotypeCaller with the "-ERC GVCF" option to obtain gVCF. All sample gVCF were then genotyped by GATK4 GenotypeGVCFs with the "-all-sites" option. We used GenMap software to calculate mappability scores with the "-K 30" and "-E 2" options, and filtered out the positions with a mappability score < 1.

For the phylogenetic analysis, we restricted our analysis to the short arm of the Y Chromosome (the first 3.4 Mb) because the vast majority of the long arm of Y is composed of amplicon sequences. We also excluded SNVs that were labeled as "LowQual" by the GATK4 HaplotypeCaller and those with read depths ≤ 3. The obtained VCF file of the Y Chromosome was converted to Fasta and Nexus files. The maximum-likelihood phylogenetic tree of the Y Chromosome was constructed using IQ-TREE2 software (Nguyen et al. 2015) with the bootstrapping of 1000 replications. According to ModelFinder (Kalyaanamoorthy et al. 2017), "TIM2+F+R2" was chosen as the best substitution model using the Bayesian Information Criterion.

Supplemental Method 3

Mitochondrial genome assembly

Complete mitochondrial sequences of all 37 samples were *de novo* assembled using GetOrganelle software (Jin et al. 2020) with maximum extension rounds set to 10 and *k*-mer parameters set to "21, 45, 65, 85, 105, 127". The qualities of assembled mitochondrial genomes were visually checked using Bandage software (Wick et al. 2015). All sample sequences were aligned using Clustal Omega with the "--auto" option. All D-Loop regions and gapped sites were removed from the alignments. A maximum-likelihood phylogenetic tree was constructed using IQ-TREE2 software (Nguyen et al. 2015) with the bootstrapping of 1000 replications. Before constructing the phylogenetic tree, we used ModelFinder software (Kalyaanamoorthy et al. 2017) implemented in IQ-TREE2 to determine the best substitution model. According to ModelFinder, "TPM2+F+I+G4" was chosen as the best substitution model using the Bayesian Information Criterion.

Supplemental Method 4

Deletion detection from the sequence data

DELLY (v.1.1.6) (Rausch et al. 2012) was used for genotyping retroelement-like *Fv1* (Friend virus susceptibility protein 1) gene deletion. Because the *Fv1* gene is located at Chr4:147,868,979–147,870,358 in Chromosome 4, we restricted the region used to detect deletions to between Chr4:145,000,000–150,000,000 to facilitate the calculations.

Supplemental Method 5

Construction of genome-wide genealogies using RELATE

We estimated the genome-wide genealogies using RELATE v.1.1.9 (Speidel et al. 2019). The input files were converted from variant call format (.vcf) to haplotype format (.haps) using the RelateFileFormats command with the "--mode ConvertFromVcf" option implemented in the RELATE package. Non-biallelic SNVs were removed from the analysis, and the *M. spretus* genome was used to assign derived mutations in *M. musculus*. A germline mutation rate of 5.7×10^{-9} per bp per generation (Milholland et al. 2017), a generation time of 1 year, and an initial effective population size parameter of 120,000, were used for estimation. We then used the EstimatePopulationSize.sh script in the RELATE package to re-estimate the effective population size changes over time. We set the threshold to remove uninformative trees at 0.5.

Reference

Andrews S. 2010. FastQC A Quality Control tool for High Throughput Sequence Data [online]. Available online at: http://www.bioinformatics.babraham.ac.uk/projects/fastqc/

Betts A, Jia PW, Dodson J. 2014. The origins of wheat in China and potential pathways for its introduction: A review. *Quat Int* **348**: 158-168.

Choi JY, Platts AE, Fuller DQ, Hsing Y-I, Wing RA, Purugganan MD. 2017. The Rice Paradox: Multiple Origins but Single Domestication in Asian Rice. *Mol Biol Evol* **34**: 969-979.

Cingolani P, Patel V, Coon M, Nguyen T, Land S, Ruden D, Lu X. 2012a. Using Drosophila melanogaster as a Model for Genotoxic Chemical Mutational Studies with a New Program, SnpSift. *Frontiers in Genetics* **3**: 35.

Cingolani P, Platts A, Wang LL, Coon M, Nguyen T, Wang L, Land SJ, Lu X, Ruden DM. 2012b. A program for annotating and predicting the effects of single nucleotide polymorphisms, SnpEff. *Fly* **6**: 80-92.

Cooke NP, Mattiangeli V, Cassidy LM, Okazaki K, Stokes CA, Onbe S, Hatakeyama S, Machida K, Kasai K, Tomioka N et al. 2021. Ancient genomics reveals tripartite origins of Japanese populations. *Sci Adv* **7**: eabh2419.

Delaneau O, Zagury J-F, Robinson MR, Marchini JL, Dermitzakis ET. 2019. Accurate, scalable and integrative haplotype estimation. *Nat Commun* **10**: 5436.

Dufresnes C, Litvinchuk SN, Borzée A, Jang Y, Li J-T, Miura I, Perrin N, Stöck M. 2016. Phylogeography reveals an ancient cryptic radiation in East-Asian tree frogs (Hyla japonica group) and complex relationships between continental and island lineages. *BMC Evol Biol* **16**: 253.

Endo E, Leipe C. 2022. The onset, dispersal and crop preferences of early agriculture in the Japanese archipelago as derived from seed impressions in pottery. *Quat Int* **623**: 35-49.

Ewels P, Magnusson M, Lundin S, Käller M. 2016. MultiQC: summarize analysis results for multiple tools and samples in a single report. *Bioinformatics* **32**: 3047-3048.

Excoffier L, Dupanloup I, Huerta-Sánchez E, Sousa VC, Foll M. 2013. Robust Demographic Inference from Genomic and SNP Data. *PLoS Genet* **9**: e1003905.

Faust GG, Hall IM. 2014. SAMBLASTER: fast duplicate marking and structural variant read extraction. *Bioinformatics* **30**: 2503-2505.

Fujio S. 2017. Rethinking the range of the Yayoi culture. *Q Archaeol* **138**: 51-54.

Fujiwara K, Kawai Y, Takada T, Shiroishi T, Saitou N, Suzuki H, Osada N. 2022a. Insights into Mus musculus Population Structure across Eurasia Revealed by Whole-Genome Analysis. *Genome Biology and Evolution* **14**: evac068.

Fujiwara K, Kawai Y, Takada T, Shiroishi T, Saitou N, Suzuki H, Osada N. 2022b. Insights into Mus musculus Population Structure across Eurasia Revealed by Whole-Genome Analysis. *Genome Biol Evol* **14**.

Hanihara K. 1991. Dual Structure Model for the Population History of the Japanese. *Japan Review*: 1-33.

Huang X, Kurata N, Wei X, Wang Z-X, Wang A, Zhao Q, Zhao Y, Liu K, Lu H, Li W et al. 2012. A map of rice genome variation reveals the origin of cultivated rice. *Nature* **490**: 497-501.

Ito T, Hayakawa T, Suzuki–Hashido N, Hamada Y, Kurihara Y, Hanya G, Kaneko A, Natsume T, Aisu S, Honda T et al. 2021. Phylogeographic history of Japanese macaques. *J Biogeogr* **48**: 1420-1431.

Jin J-J, Yu W-B, Yang J-B, Song Y, dePamphilis CW, Yi T-S, Li D-Z. 2020. GetOrganelle: a fast and versatile toolkit for accurate de novo assembly of organelle genomes. *Genome Biol* **21**: 241.

Kakioka R, Kokita T, Tabata R, Mori S, Watanabe K. 2012. The origins of limnetic forms and cryptic divergence in Gnathopogon fishes (Cyprinidae) in Japan. *Environ Biol Fishes* **96**: 631-644.

Kalyaanamoorthy S, Minh BQ, Wong TKF, von Haeseler A, Jermiin LS. 2017. ModelFinder: fast model selection for accurate phylogenetic estimates. *Nat Methods* **14**: 587-589.

Kanzawa-Kiriyama H, Jinam TA, Kawai Y, Sato T, Hosomichi K, Tajima A, Adachi N, Matsumura H, Kryukov K, Saitou N et al. 2019. Late Jomon male and female genome sequences from the Funadomari site in Hokkaido, Japan. *Anthropol Sci* **127**: 83-108.

Kato D-i, Suzuki H, Tsuruta A, Maeda J, Hayashi Y, Arima K, Ito Y, Nagano Y. 2020. Evaluation of the population structure and phylogeography of the Japanese Genji firefly, Luciola cruciata, at the nuclear DNA level using RAD-Seq analysis. *Scientific Reports* **10**: 1533.

Leipe C, Long T, Sergusheva EA, Wagner M, Tarasov PE. 2019. Discontinuous spread of millet agriculture in eastern Asia and prehistoric population dynamics. *Sci Adv* **5**: eaax6225.

Li H, Durbin R. 2009. Fast and accurate short read alignment with Burrows–Wheeler transform. *Bioinformatics* **25**: 1754-1760.

Li Y, Fujiwara K, Osada N, Kawai Y, Takada T, Kryukov AP, Abe K, Yonekawa H, Shiroishi T, Moriwaki K et al. 2021. House mouse Mus musculus dispersal in East Eurasia inferred from 98 newly determined complete mitochondrial genome sequences. *Heredity (Edinb)* **126**: 132-147.

Liu EY, Morgan AP, Chesler EJ, Wang W, Churchill GA, Pardo-Manuel de Villena F. 2014. High-Resolution Sex-Specific Linkage Maps of the Mouse Reveal Polarized Distribution of Crossovers in Male Germline. *Genetics* **197**: 91-106.

McKenna A, Hanna M, Banks E, Sivachenko A, Cibulskis K, Kernytsky A, Garimella K, Altshuler D, Gabriel S, Daly M et al. 2010. The Genome Analysis Toolkit: A MapReduce framework for analyzing next-generation DNA sequencing data. *Genome Res* **20**: 1297-1303.

Milholland B, Dong X, Zhang L, Hao X, Suh Y, Vijg J. 2017. Differences between germline and somatic mutation rates in humans and mice. *Nat Commun* **8**: 15183.

Nguyen L-T, Schmidt HA, von Haeseler A, Minh BQ. 2015. IQ-TREE: A Fast and Effective Stochastic Algorithm for Estimating Maximum-Likelihood Phylogenies. *Mol Biol Evol* **32**: 268-274.

Ning C, Li T, Wang K, Zhang F, Li T, Wu X, Gao S, Zhang Q, Zhang H, Hudson MJ et al. 2020. Ancient genomes from northern China suggest links between subsistence changes and human migration. *Nat Commun* **11**: 2700.

Okamoto T, Hikida T. 2012. A new cryptic species allied to Plestiodon japonicus (Peters, 1864) (Squamata: Scincidae) from eastern Japan, and diagnoses of the new species and two parapatric congeners based on morphology and DNA barcode. *Zootaxa* **3436**.

Osada N, Kawai Y. 2021. Exploring models of human migration to the Japanese archipelago using genome-wide genetic data. *Anthropol Sci* **129**: 45-58.

Pockrandt C, Alzamel M, Iliopoulos CS, Reinert K. 2020. GenMap: ultra-fast computation of genome mappability. *Bioinformatics* **36**: 3687-3692.

Rausch T, Zichner T, Schlattl A, Stutz AM, Benes V, Korbel JO. 2012. DELLY: structural variant discovery by integrated paired-end and split-read analysis. *Bioinformatics* **28**: i333-i339.

Speidel L, Forest M, Shi S, Myers SR. 2019. A method for genome-wide genealogy estimation for thousands of samples. *Nat Genet* **51**: 1321-1329.

Tominaga K, Nakajima J, Watanabe K. 2015. Cryptic divergence and phylogeography of the pike gudgeon Pseudogobio esocinus (Teleostei: Cyprinidae): a comprehensive case of freshwater phylogeography in Japan. *Ichthyol Res* **63**: 79-93.

Tsuchiya K. 1974. Cytological and biochemical studies of Apodemus speciosus group in Japan. *Journal of the Mammalogical Society of Japan* **6**: 67-87.

Van der Auwera GA, Carneiro MO, Hartl C, Poplin R, del Angel G, Levy-Moonshine A, Jordan T, Shakir K, Roazen D, Thibault J et al. 2002. From FastQ Data to High-Confidence Variant Calls: The Genome Analysis Toolkit Best Practices Pipeline. In *Current Protocols in Bioinformatics*, doi:10.1002/0471250953.bi1110s43. John Wiley & Sons, Inc.

Watanabe Y, Isshiki M, Ohashi J. 2021. Prefecture-level population structure of the Japanese based on SNP genotypes of 11,069 individuals. *J Hum Genet* **66**: 431-437.

Wick RR, Schultz MB, Zobel J, Holt KE. 2015. Bandage: interactive visualization of de novo genome assemblies. *Bioinformatics* **31**: 3350-3352.

Yang MA, Fan X, Sun B, Chen C, Lang J, Ko Y-C, Tsang C-h, Chiu H, Wang T, Bao Q et al. 2020. Ancient DNA indicates human population shifts and admixture in northern and southern China. *Science* doi:10.1126/science.aba0909: eaba0909.

Yonekawa H, Moriwaki K, Gotoh O, Miyashita N, Matsushima Y, Shi LM, Cho WS, Zhen XL, Tagashira Y. 1988. Hybrid origin of Japanese mice "Mus musculus molossinus": evidence from restriction analysis of mitochondrial DNA. *Mol Biol Evol* **5**: 63-78.
