# Supplementary material for: Inference of selective forces on house mouse genomes during secondary contact in East Asia
Source: Genome Res. 2024 Mar;34(3):366–75. doi: 10.1101/gr.278828.123 (PMC11067880; doi:10.1101/gr.278828.123)
Supplement: Supplement 2 [file Supplemental_Figures.pdf]

Supplemental Materials for

Inference of selective force on house mouse genome during secondary contact in East Asia

Kazumichi Fujiwara, Shunpei Kubo, Toshinori Endo, Toyoyuki Takada, Toshihiko Shiroishi, Hitoshi Suzuki, Naoki Osada

Table of Contents:

1 **Supplemental Figure S1**: Principal component analysis (PCA) of wild house mouse using 163 *Mus musculus* samples

2 **Supplemental Figure S2**: ADMIXTURE plot of *Mus musculus* samples using autosome data

3 **Supplemental Figure S3**: The maximum likelihood tree of mitochondrial genomes

4 **Supplemental Figure S4**: The maximum likelihood tree of the short arm of the Y Chromosomes

5 **Supplemental Figure S5**: Correlation between *Slx* and *Sly* copy numbers in different subspecies of house mouse

6 **Supplemental Figure S6**: Fixation rates of X and Y Chromosomes within 0.1N generations, considering changes in male-to-female birth ratio (α) and fertility reduction due to sex-ratio distortion (R_{XY})

7 **Supplemental Figure S7**: Trajectories of allele frequency of introgressed alleles

8 **Supplemental Figure S8**: Fixation rates of X and Y Chromosomes within 0.1N generations, considering changes in recombination rate per generation between SD and XHI loci (r), with $R_{XA} = 1$

9 **Supplemental Figure S9**: The demographic model to estimate the population genetic parameters

10 **Supplemental Figure S10**: Expected and observed folded site frequency spectrum for the Japanese population

11 **Supplemental Figure S11**: Gene density and recombination rate comparisons for *castaneus*-enriched windows

12 **Supplemental Figure S12**: Functional enrichment analysis of genes with *castaneus*-ancestry bias, analyzing a total of 842 genes

13 **Supplemental Figure S13**: Segregation patterns of nonsynonymous SNVs within the *Irgm1* gene of wild house mice

14 **Supplemental Figure S14**: segregation patterns of nonsynonymous SNVs within the *Irgm2* gene of wild house mice

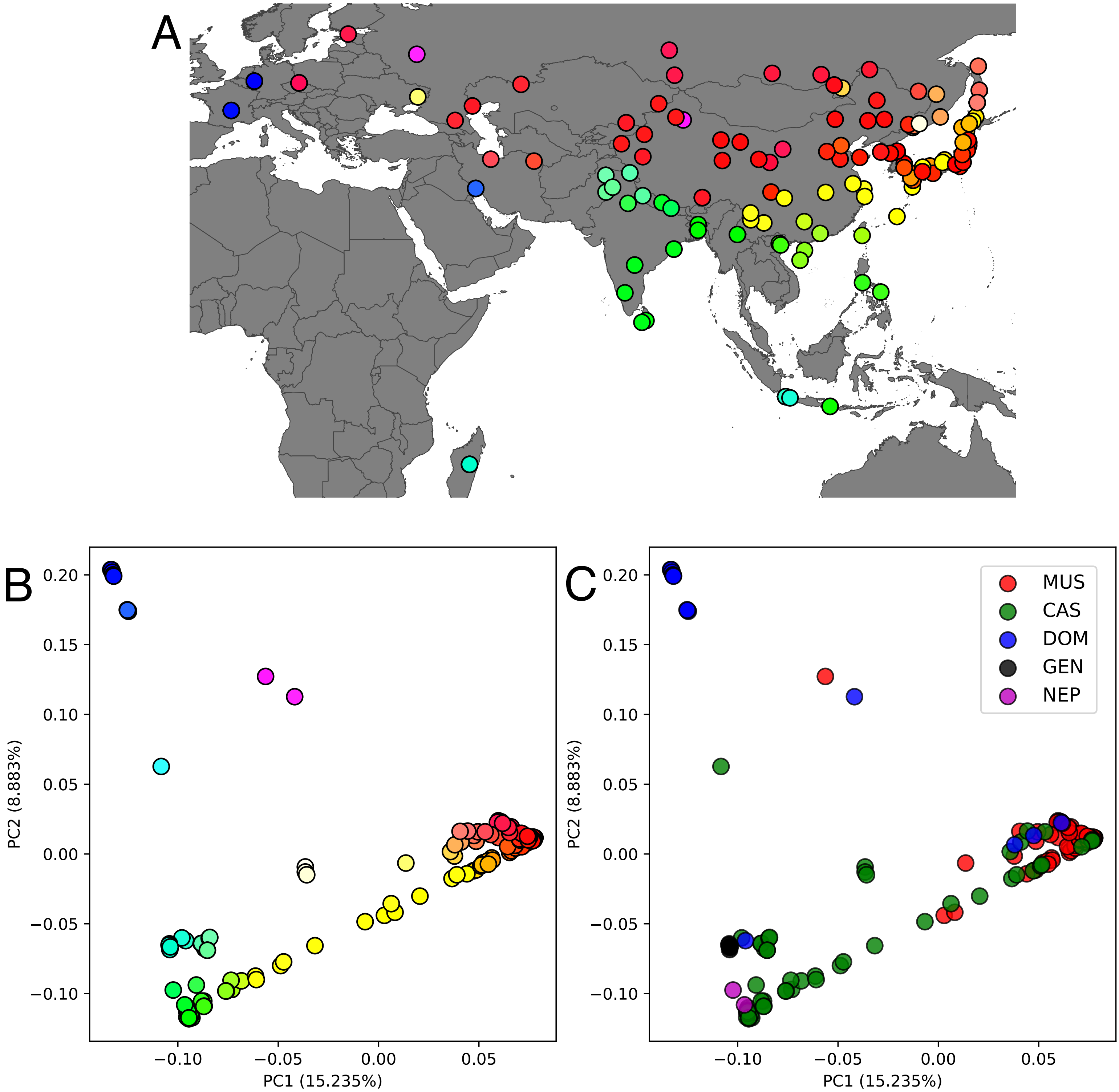

Supplemental Figure S1.

Principal component analysis (PCA) of wild house mouse using 163 *Mus musculus* samples. **(A)** Geographic map of the samples collected used in this study. Each circle represents an individual, and the color of the circle corresponds to the color used in panel (B). **(B)** Plot results of principal component analysis using autosomes of wild house mice. Red, green, and blue circles correspond to *musculus*, *castaneus*, and *domesticus* genetic components, respectively. The circles showing the intermediate color of each genetic component indicate the hybridization of each genetic component. The proportion of variance for each principal component (PC) is shown in each axis labels. **(C)** PCA results shown in panel (B) with mitochondrial haplogroup labels. The labels with MUS, CAS, and DOM represent the *musculus*, *castaneus*, and *domesticus* haplogroups, respectively. In addition, GEN and NEP represents distinct haplogroup in Madagascar and distinct haplogroup in Nepal, respectively.

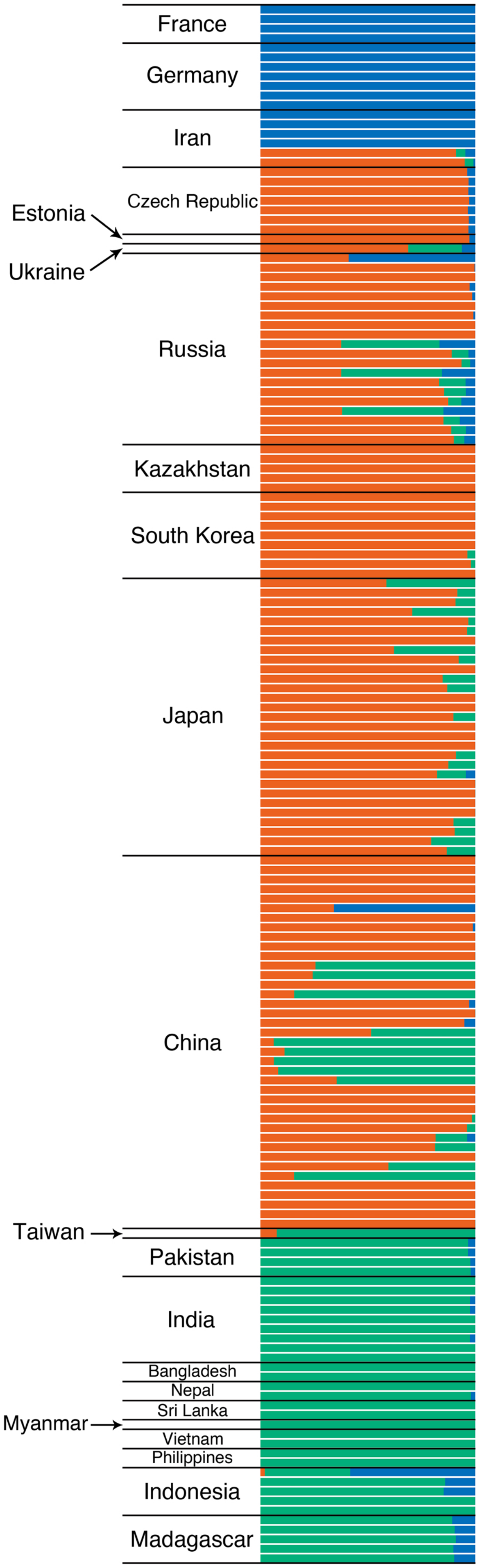

Supplemental Figure S2.

ADMIXTURE plot of *Mus musculus* samples using autosome data. Each bar represents each individual, and the color ratio represents the proportion of genetic elements. The results are shown for K=3, where red, green and blue colors represent the genetic elements of *musculus*, *castaneus* and *domesticus*, respectively.

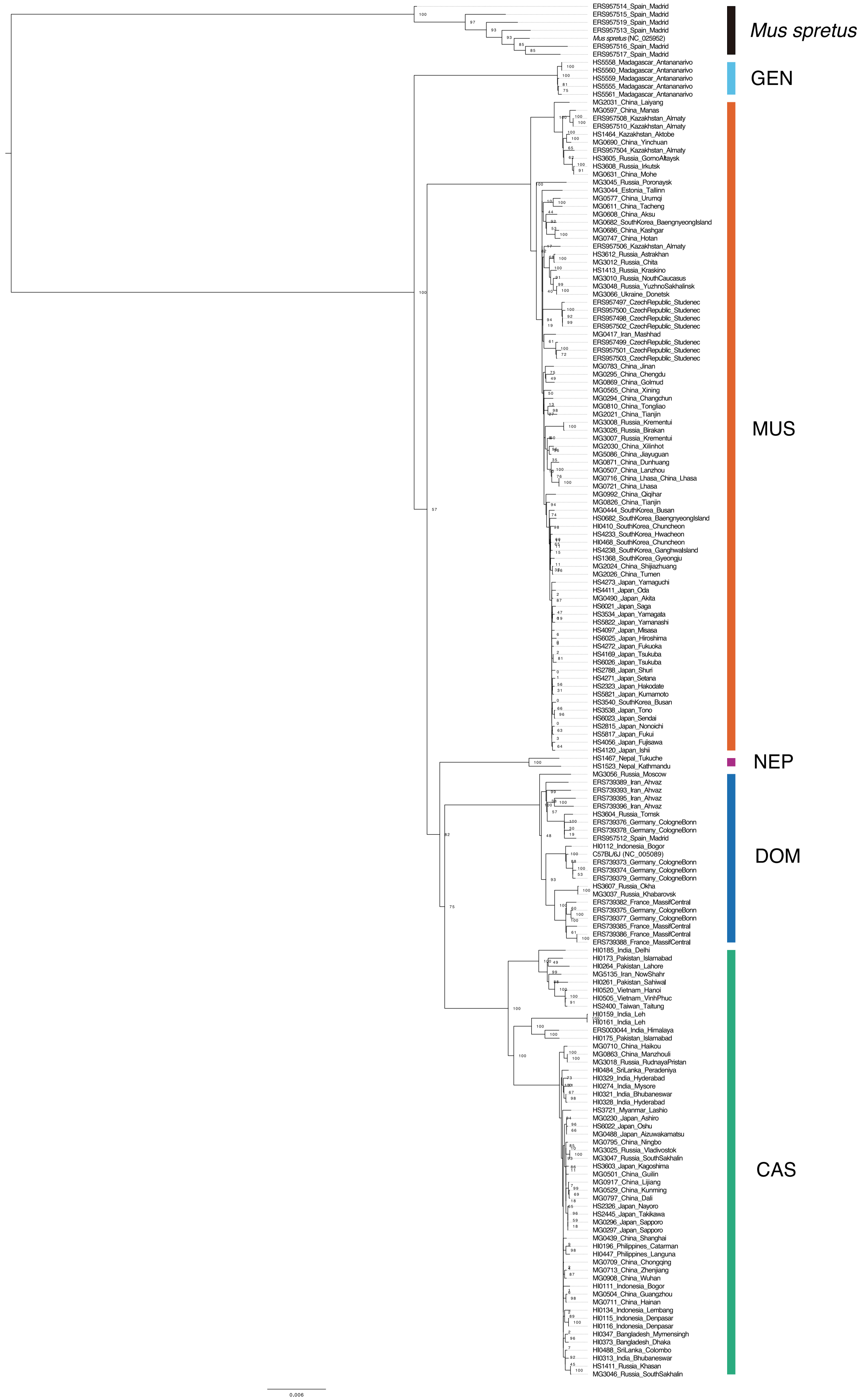

Supplemental Figure S3.

The maximum likelihood tree of mitochondrial genomes. The phylogenetic tree contains the mitochondrial sequence of 170 samples used in this study and additionally includes the mitochondrial reference sequences of *Mus spretus* (NC_025952) and *Mus musculus* (C57BL/6J strain, NC_005089). The substitution model (TPM2+F+I+G4) was determined as the best fit model by ModelFinder implemented in IQ-TREE2. Each captioned colored line indicates the corresponding clade of *Mus musculus* subspecies-derived haplotypes. The node labels exhibit support assigned by bootstrapping for 1000 replications. The labels with CAS, DOM, and MUS represent *castaneus, domesticus,* and *musculus* haplogroups, respectively. GEN and NEP labels show distinct haplogroups in Madagascar and Nepal, respectively.

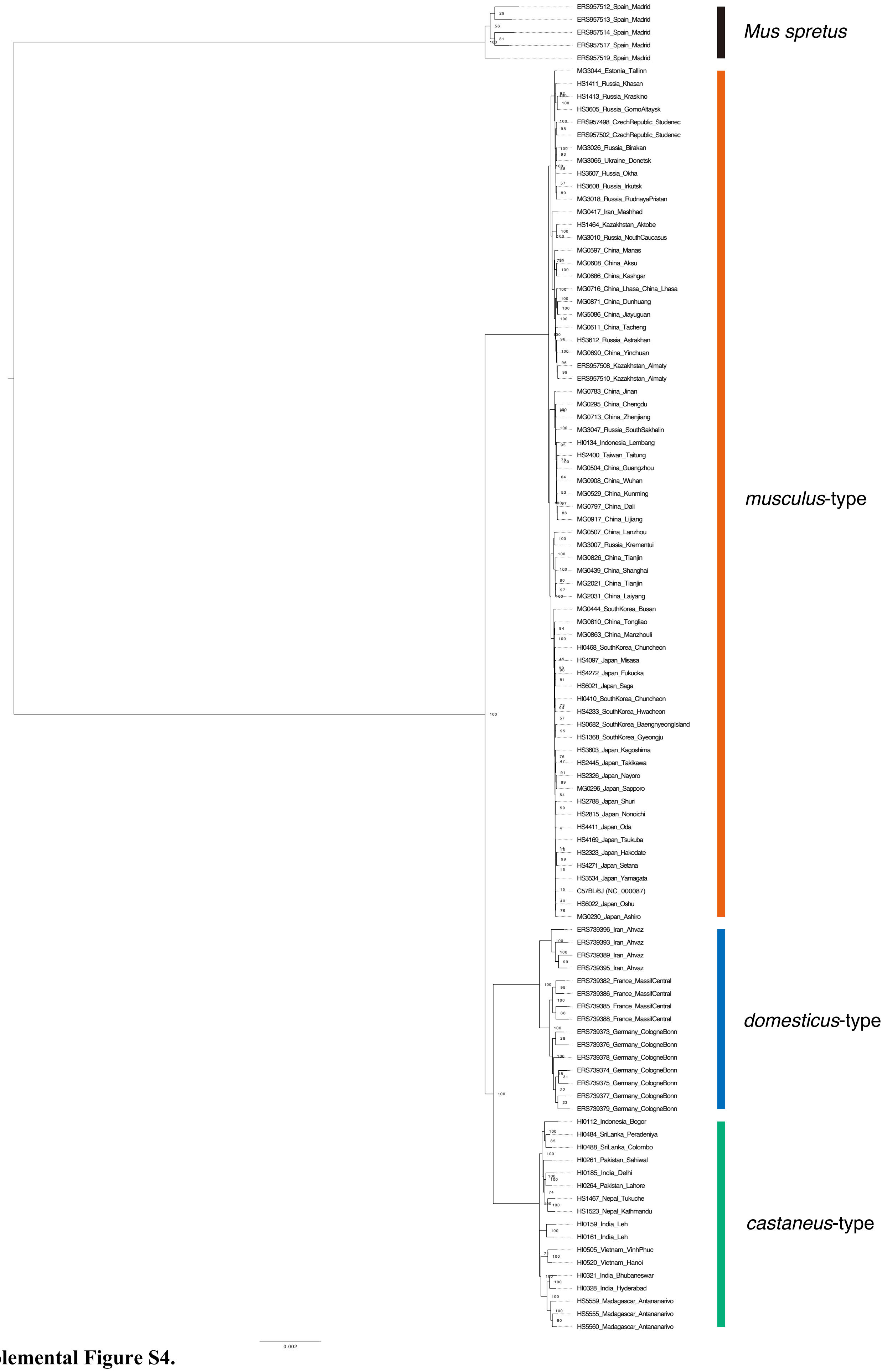

Supplemental Figure S4.

The maximum likelihood tree of the short arm of the Y Chromosome. The phylogenetic tree contains the Y Chromosome sequences of 103 male samples used in this study and additionally includes the Y Chromosome reference sequence of *Mus musculus* (C57BL/6J strain, NC_000087). The substitution model (TIM2+F+R2) was determined as the best fit model by ModelFinder implemented in IQ-TREE2. Each captioned colored line indicates the corresponding clade of *Mus musculus* subspecies-derived haplotype. The node labels exhibit support assigned by bootstrapping for 1000 replications.

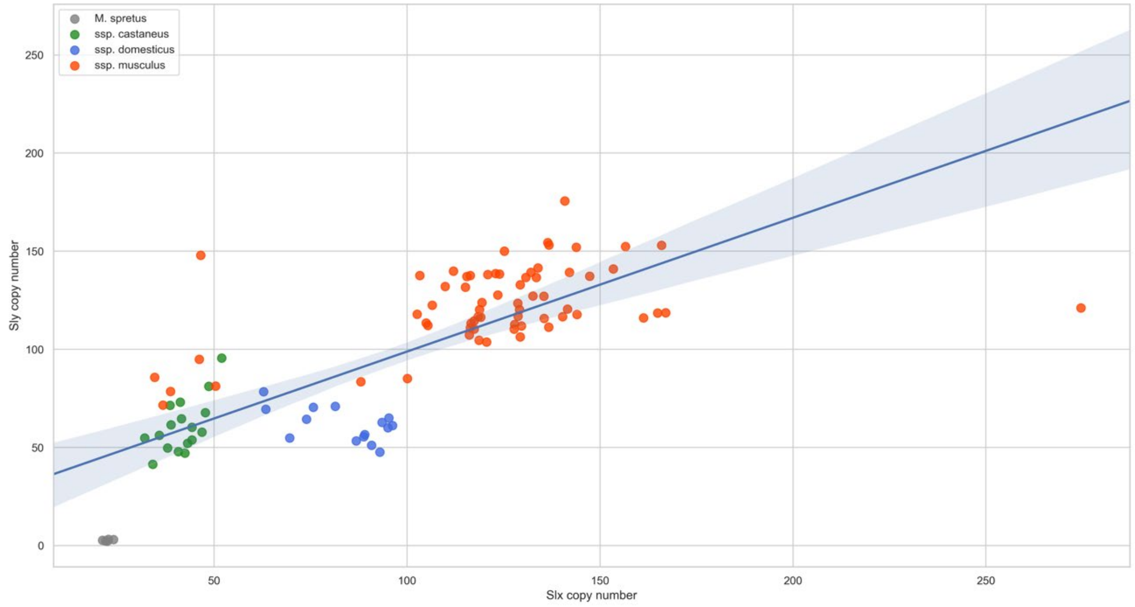

Supplemental Figure S5

Correlation between *Slx* and *Sly* copy numbers in different subspecies of house mouse. The *x*-axis represents the *Slx* copy number, while *y*-axis represents the *Sly* copy number. Each point on the plot corresponds to an individual sample, colored by subspecies: *M. spretus* (gray), ssp. *castaneus* (green), ssp. *domesticus* (blue), and ssp. *musculus* (red). A linear regression line has been fitted to the data points, with the surrounding shaded area indicating the 95% confidence interval for the regression line (R^2 = 0.60).

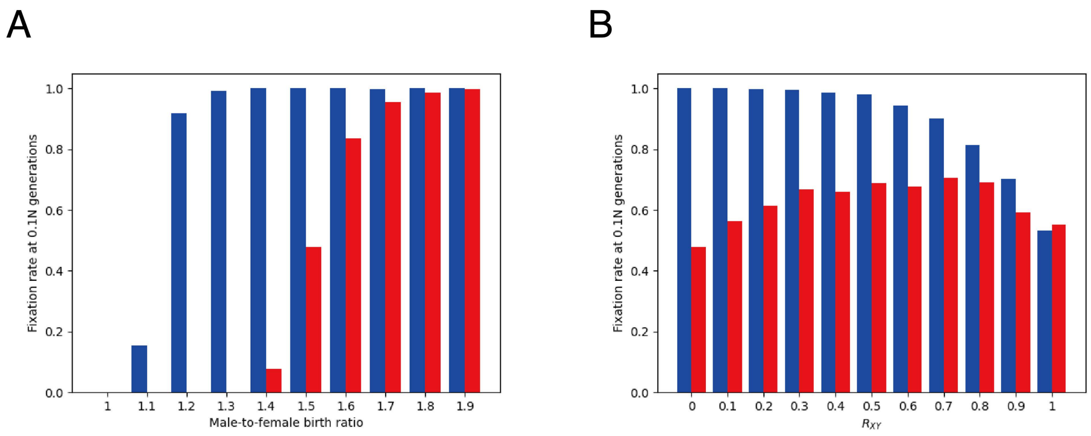

Supplemental Figure S6

Fixation rates of X and Y Chromosomes within 0.1*N* generations, considering changes in male-to-female birth ratio (α, panel A) and fertility reduction due to sex-ratio distortion (R_{XY}, panel B). Parameter setting in the panel A and B is, [$R_{AA} = 0$, $R_{XA} = 0$, $R_{MA} = 0$, $R_{XY} = 0$, $Nm = 0$] and [$R_{AA} = 0$, $R_{XA} = 0$, $R_{MA} = 0$, $\alpha = 1.5$, $Nm = 0$], respectively. The blue and red bars represent the fixation rate of introgressed Y and X Chromosomes after 0.1*N* generations, respectively.

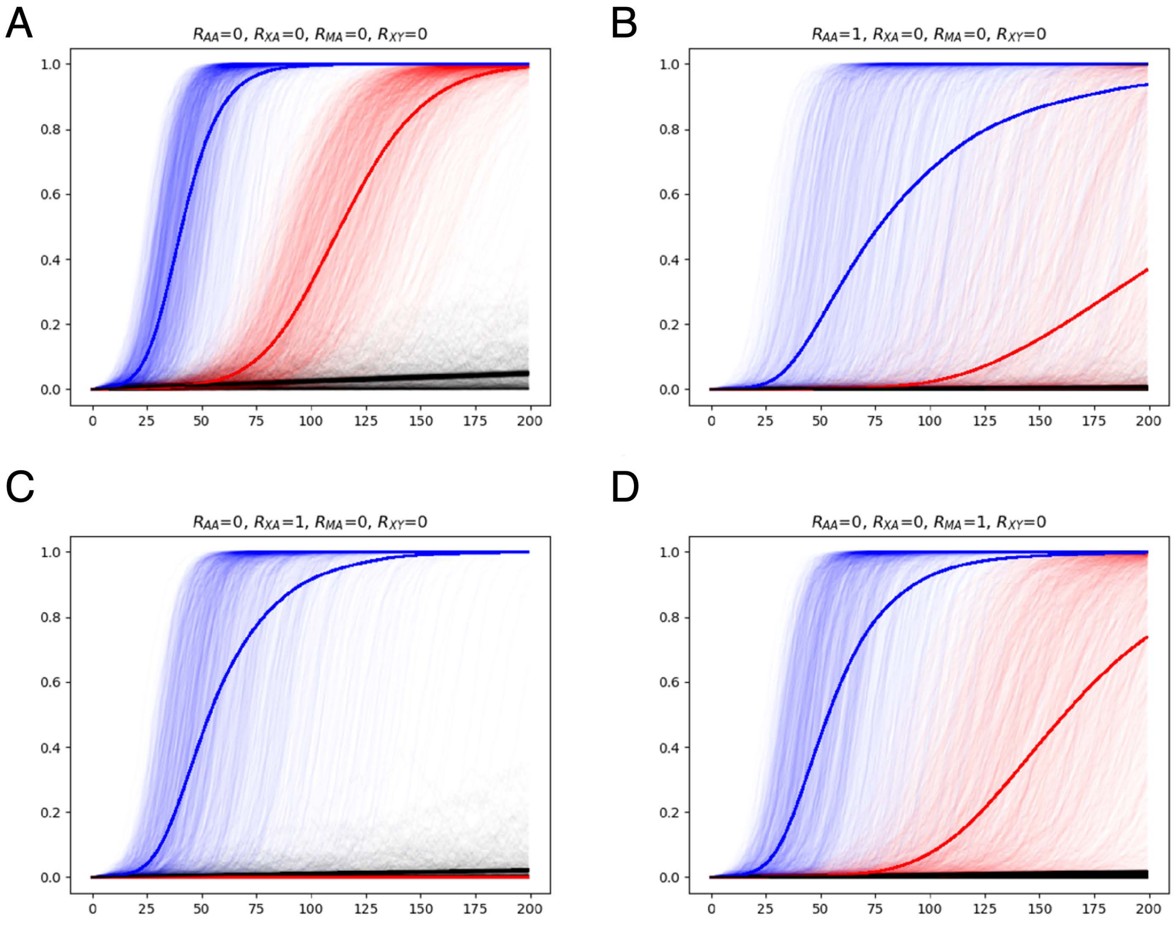

Supplemental Figure S7

Trajectories of allele frequency of introgressed alleles. The red, blue, and black lines show the allele frequency at introgressed X-chromosomal, Y-chromosomal, and mitochondrial genomes, respectively. The thin lines represent the trajectories of single simulation, and the thick lines show the average allele frequencies among 1000 trials. The parameter setting of each panel is shown in the top of plot. Sex-ratio distortion parameter α was 1.5 and recombination rate between SD and XHI loci was zero. See Supplemental Note 1 for detailed methods and results.

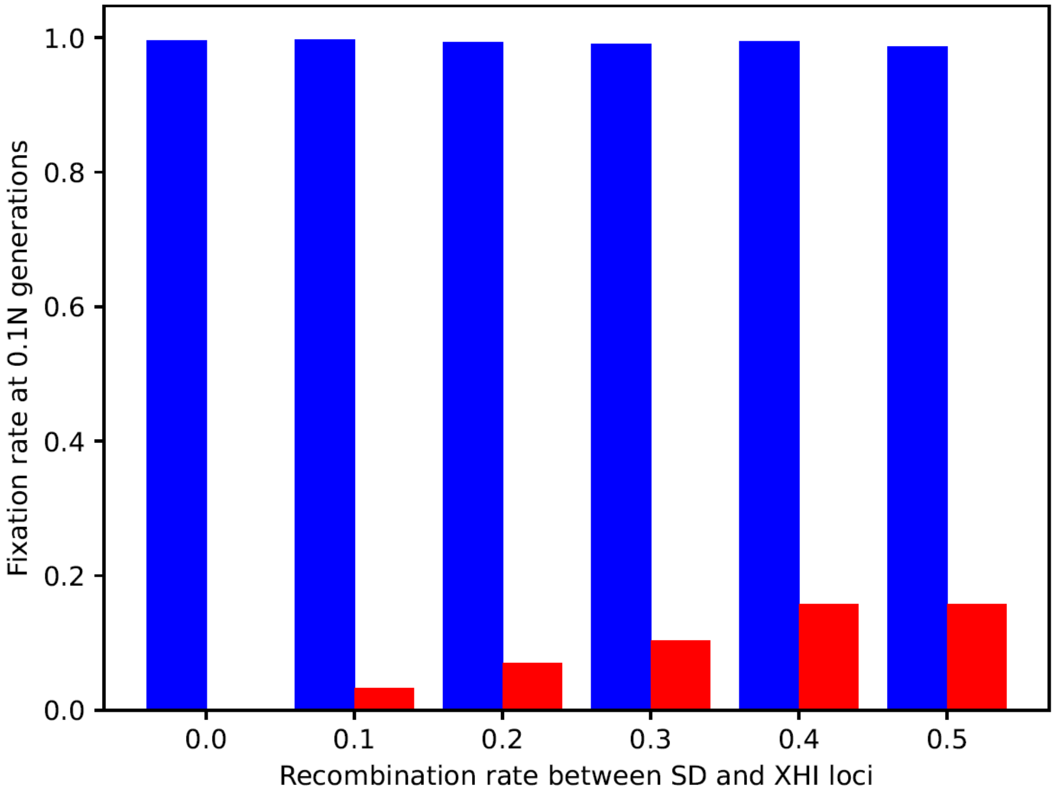

Supplemental Figure S8

Fixation rates of X and Y Chromosomes within $0.1N$ generations, considering changes in recombination rate per generation between SD and XHI loci (r), with $R_{XA} = 1$. Note that SD locus is in between two XHI loci and the SD and XHI loci are separated with the distance equivalent to r. Other parameter setting is following, $R_{AA} = 0$, $R_{MA} = 0$, $\alpha = 1.5$, and $Nm = 1$. The blue and red bars represent the fixation rate of introgressed Y and X Chromosomes, respectively.

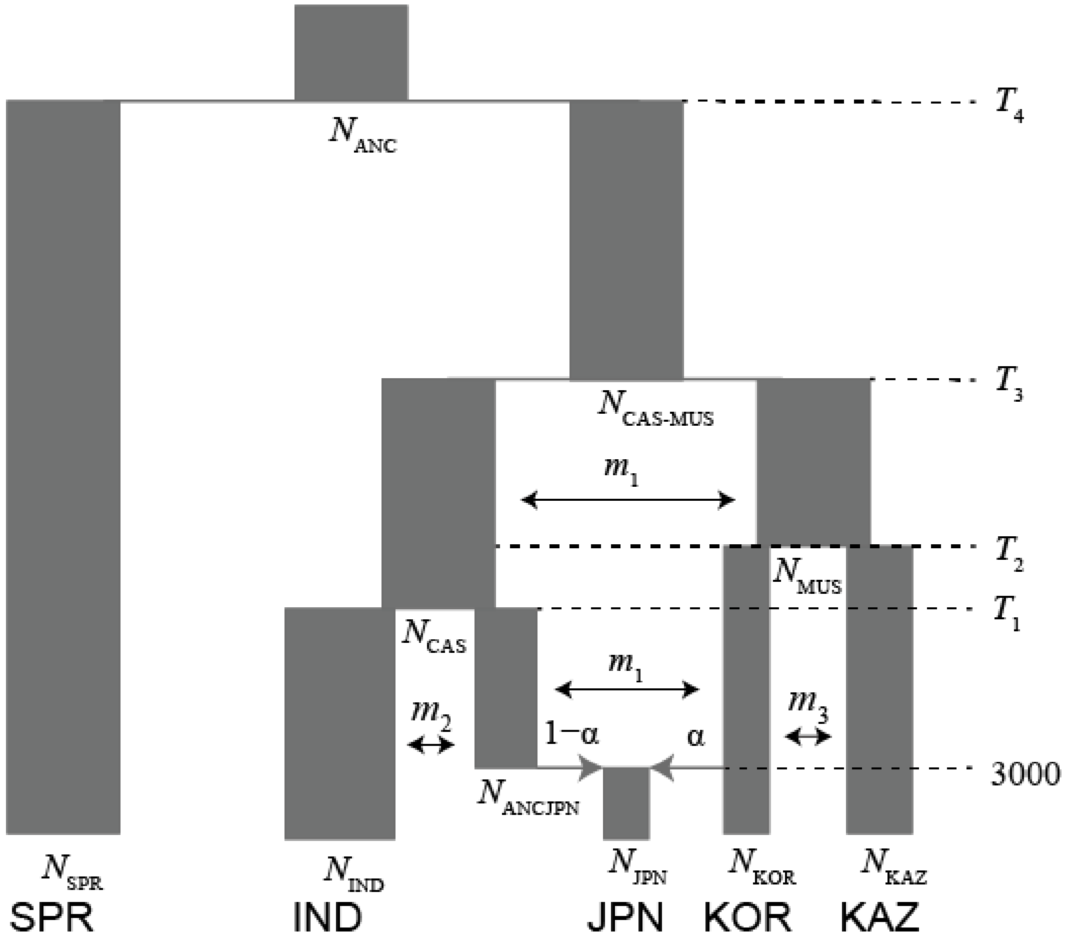

Supplemental Figure S9

The demographic model we used to estimate the population genetic parameters. The parameters were estimated using the site frequency spectrum data of the five populations shown in the figure.

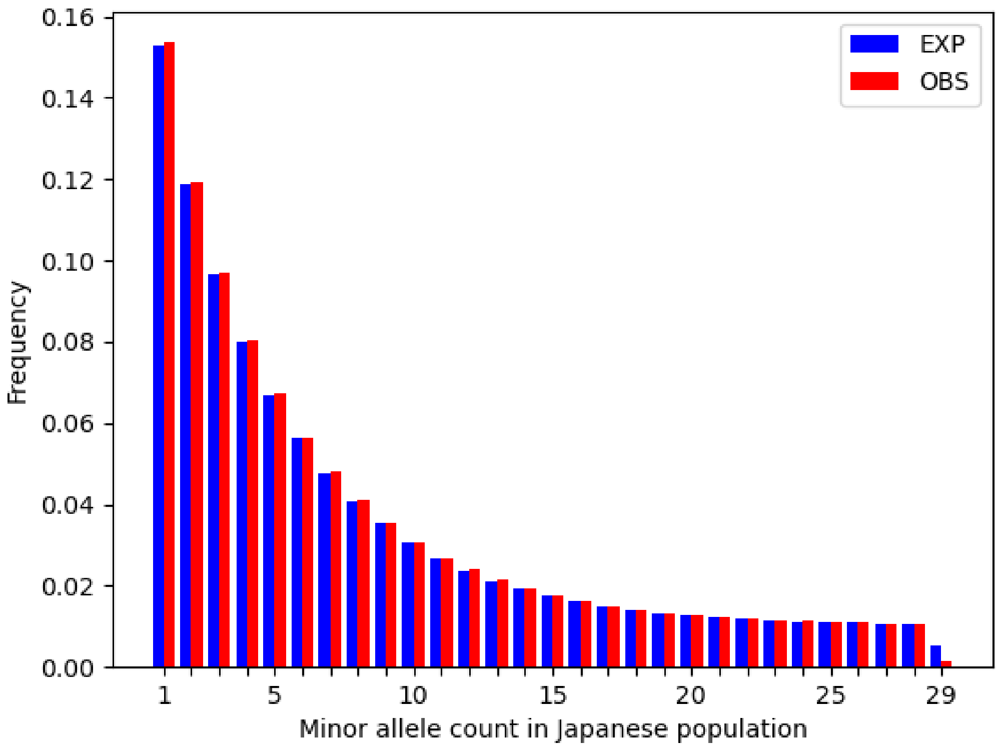

Supplemental Figure S10

Expected and observed folded site frequency spectrum for the Japanese population. The blue bars indicate the expected frequencies, derived from parameter optimization using fastsimcoal2, while the red bars represent the observed frequencies.

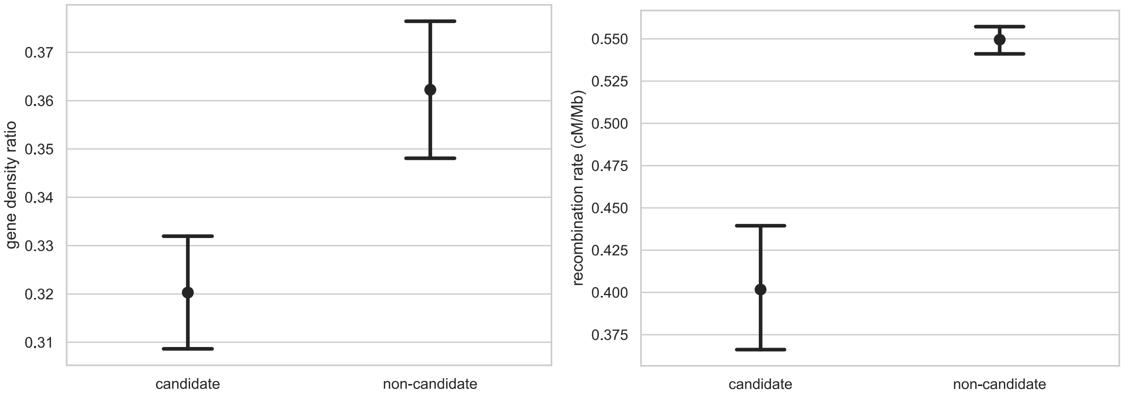

Supplemental Figure S11

Gene density and recombination rate comparisons for *castaneus*-enriched windows. The term "candidate" denotes windows with a high concentration of *castaneus* ancestry, as opposed to "non-candidate" windows in the Japanese population genome. For gene density (left panel), error bars show the standard deviation derived from 1000 bootstrap samples, each consisting of 1000 windows chosen at random. In terms of recombination rate (right panel), the depicted bars outline the 95% confidence intervals.

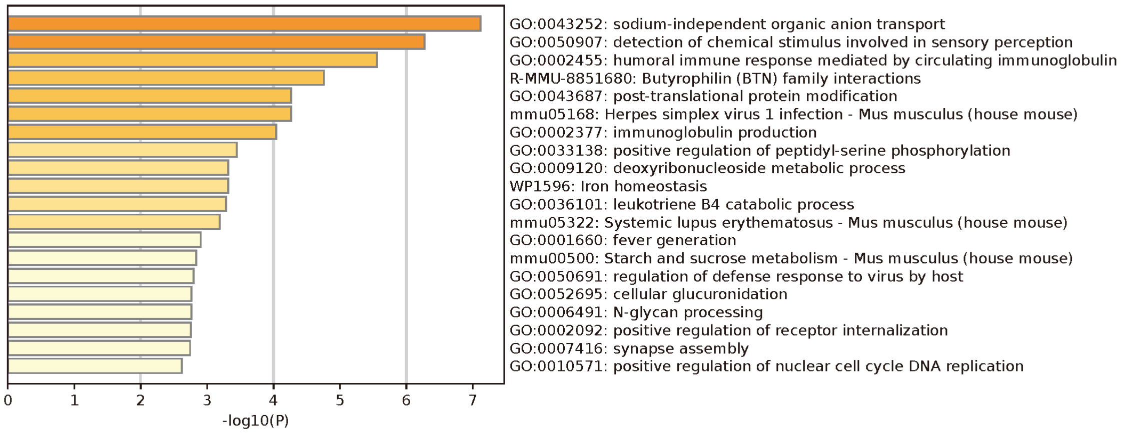

Supplemental Figure S12

Functional enrichment analysis of genes with *castaneus*-ancestry bias, analyzing a total of 842 genes. Gene categories with a *p*-value less than 0.05 are presented.

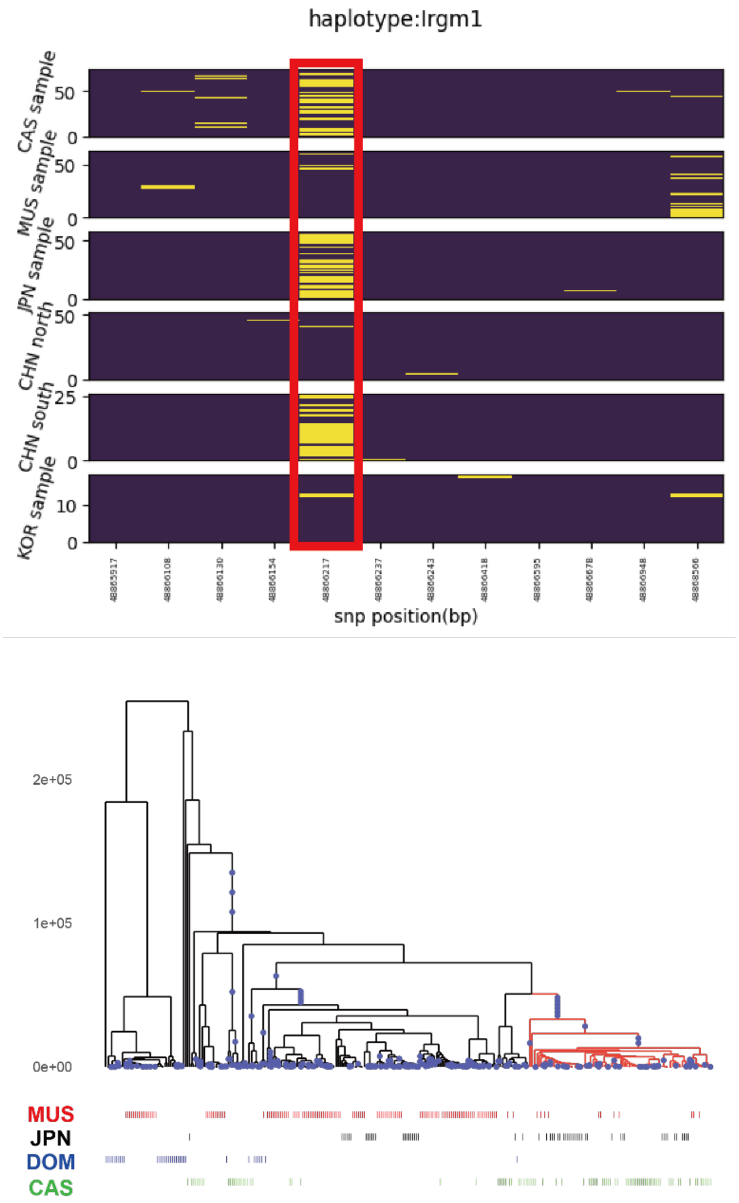

Supplemental Figure S13

The upper panel of this figure displays the segregation patterns of nonsynonymous SNVs within the *Irgm1* gene of wild house mice. CAS denotes samples from the subspecies castaneus, excluding those from southern China (CHN South). MUS refers to subspecies musculus samples, but excludes those from Korea (KOR) and northern China (CHN North). JPN denotes Japanese samples, and DOM represents the subspecies domesticus. The site marked by a red rectangle is the focal site.

In the lower panel, the inferred genealogy around this focal site is depicted. Branches carrying the derived mutation at this focal site are highlighted in red. Blue dots indicate mutations on branches. The sample labels are provided at the bottom of the tree for reference.

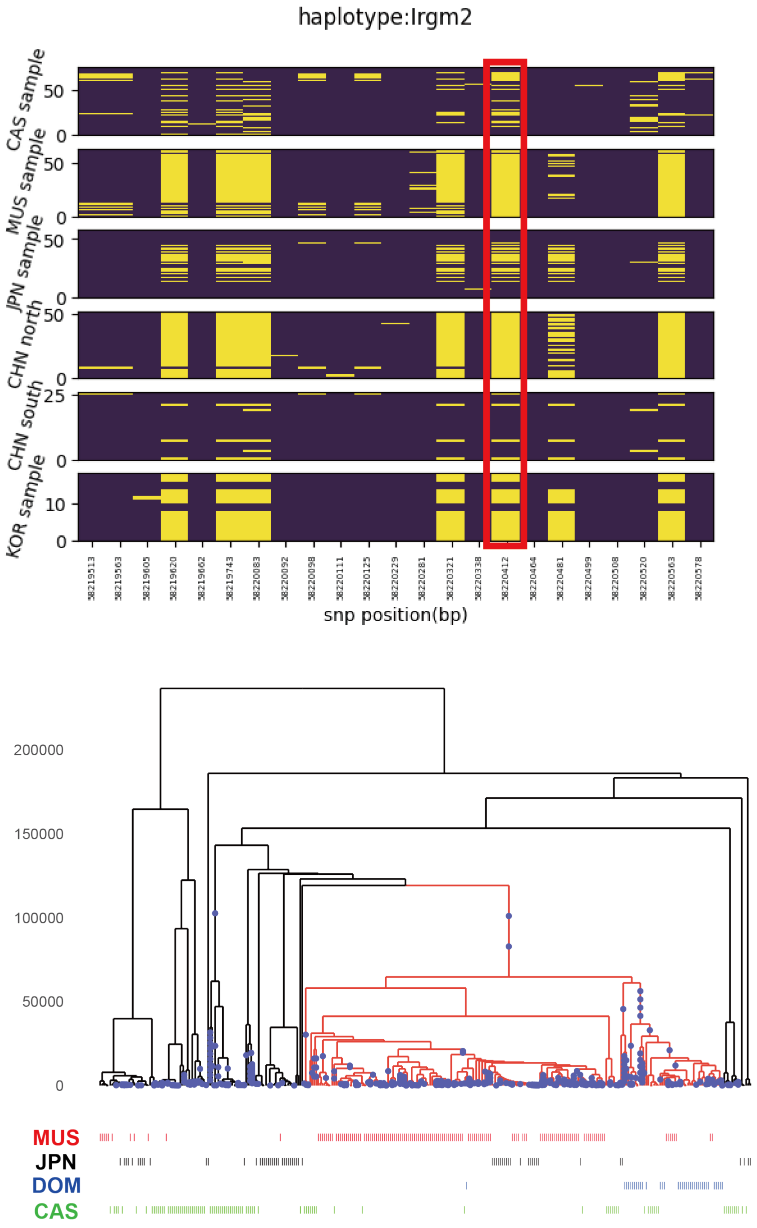

Supplemental Figure S14

The upper panel of this figure displays the segregation patterns of nonsynonymous SNVs within the *Irgm2* gene of wild house mice. CAS denotes samples from the subspecies castaneus, excluding those from southern China (CHN South). MUS refers to subspecies musculus samples, but excludes those from Korea (KOR) and northern China (CHN North). JPN denotes Japanese samples, and DOM represents the subspecies domesticus. The site marked by a red rectangle is the focal site.

In the lower panel, the inferred genealogy around this focal site is depicted. Branches carrying the derived mutation at this focal site are highlighted in red. Blue dots indicate mutations on branches. The sample labels are provided at the bottom of the tree for reference.
